# *In vitro* bioanalytical evaluation of removal efficiency for bioactive chemicals in Swedish wastewater treatment plants

**DOI:** 10.1038/s41598-019-43671-z

**Published:** 2019-05-09

**Authors:** Johan Lundqvist, Geeta Mandava, Sebastian Lungu-Mitea, Foon Yin Lai, Lutz Ahrens

**Affiliations:** 1 Department of Biomedical Sciences and Veterinary Public Health, Swedish University of Agricultural Sciences, Box 7028, SE-750 07 Uppsala, Sweden; 2 Department of Aquatic Sciences and Assessment, Swedish University of Agricultural Sciences, Box 7050, SE-750 07 Uppsala, Sweden

**Keywords:** Environmental impact, Endocrinology

## Abstract

Chemical contamination of wastewater is a problem of great environmental concern, as it poses a hazard to both the ecosystem and to human health. In this study, we have performed a bioanalytical evaluation of the presence and removal efficiency for bioactive chemicals in wastewater treatment plants (WWTPs), using *in vitro* assays for toxicity endpoints of high relevance for human health. Water samples were collected at the inlet and outlet of five Swedish WWTPs, all adopting a treatment technology including pretreatment, primary treatment (sedimenation), seconday treatment (biological processes), post-sedimentation, and sludge handling. The water samples were analyzed for cytotoxicity, estrogenicity, androgenicity, aryl hydrocarbon receptor (AhR) activity, oxidative stress response (Nrf2) and the ability to activate NFĸB (nuclear factor kappa-light-chain-enhancer of activated B cells) signaling. We observed clear androgenic and estrogenic activities in all inlet samples. Androgenic and estrogenic activities were also observed in all outlet samples, but the activities were lower than the respective inlet sample. AhR activity was observed in all samples, with higher activities in the inlet samples compared to the outlet samples. The removal efficiency was found to be high for androgenic (>99% for two plants and 50–60% for two plants) and estrogenic (>90% for most plants) compounds, while the removal efficiency for AhR-inducing compounds was 50–60% for most plants and 16% for one plant.

## Introduction

Wastewater contaminated with a wide range of chemicals can be an environmental problem of great concern for the aquatic system where the treated wastewater is discharged, especially if the chemicals are not targeted during the wastewater treatment process^1,2^. Furthermore, many surface water systems are used as a source for drinking water production, and the potential discharge of bioactive chemicals from wastewater treatment plants (WWTPs) can thereby also pose a potential threat to human health, due to exposure via drinking water^3–5^. Approximately 50% of the drinking water in Sweden is produced from surface water, and a large portion of these surface waters are affected by WWTP effluents. The removal efficiency of chemicals in wastewater treatment processes have traditionally been monitored using chemical analysis^6,7^. However, due to the large number of possible contaminants, it is not possible to analyze all pollutants with such strategy. Bioanalytical methods can be valuable tools to overcome this limitation and analyze the removal efficiency of all bioactive chemicals during wastewater treatment^8,9^. It has repeatedly been reported that analyzed chemicals only explain a relatively small fraction of the observed bioactivities in environmental water samples^1,2,10,11^, highlighting the need for effect-based approaches to fully understand the presence of known and unknown chemicals in the aquatic environment and to assess the removal efficiency of chemical pollutants during wastewater treatment.

Bioanalytical methods, such as *in vitro* bioassays based on human cells designed to respond to specific toxicity mechanisms, has been used for water quality assessments of wastewater, drinking water and environmental waters^1–5,8,9,12–22^. When using bioanalytical methods for water quality assessment, it is important to select a panel of suitable bioassays covering toxic activities of a broad range of chemicals. It has been reported that the most responsive and health-relevant endpoints for water quality testing were related to xenobiotic metabolism, hormone-mediated modes of action and genotoxicity/oxidative stress^12^. There are a wide range of chemicals that can activate these endpoints. For some endpoints, a large portion of the environmentally relevant effects can be expected to come from a few compounds (*e.g*. the estrogen and androgen receptor signaling pathways where natural hormones and drug residues can explain a large part of the effect) while other endpoints (*e.g*. oxidative stress) are activated by a broad range of compounds and the known pollutants can only explain a very small fraction of the observed effects^2,11^.

Previous bioanalytical evaluations of the toxicity removal efficiency during wastewater treatment has revealed differences in the removal efficiency depending on which toxicity end-point that was studied^8,15,23–25^.

In this study, we have performed an effect-based assessment of the removal efficiency for bioactive chemical pollutants in five Swedish WWTPs, all adopting a treatment technology including pretreatment (grit removal, settlement of sand or grit), primary treatment (sedimenation), seconday treatment (biological processes), post-sedimentation, and sludge handling. Wastewater samples were collected at the inlet and outlet of the WWTPs and each sample was analyzed for cytotoxicity, estrogenicity, androgenicity, aryl hydrocarbon receptor activity, oxidative stress response (Nrf2) and the ability to activate NFĸB (nuclear factor kappa-light-chain-enhancer of activated B cells) signaling. With this approach, we were able to evaluate how efficiently the bioactive chemicals, both known and unknown, were removed from the water for a broad range of health-relevant toxicity endpoints.

## Materials and Methods

### Water sample collection

Water samples were collected at five Swedish WWTPs in March and April 2018. The WWTPs were selected as they are representative of many large-scale wastewater treatment facilities in Sweden. Samples covered both the inlet (untreated) water and the corresponding outlet (treated) water at each WWTP. A flow-proportional sampling mode was used for sample collection over a time of 24–144 h to integrate short-term fluctuations in the water composition. The treatment steps were the same for all studied WWTPs. These involved pretreatment (grit removal, settlement of sand or grit), primary treatment (sedimenation), seconday treatment (biological processes), post-sedimentation, and sludge handling (sludge thickener, digestion chamber). Details on the WWTPs are presented in Table 1, with additional information provided in Table SI-1. Water samples were collected in 12 L stainless steel containers, transported to the laboratory and stored at +4 °C awaiting sample preparation.

**Table 1:** Information on the WWTPs included in this study.

Location	City	Population equivalents	Average flow (m^3^/24 h)	Industrial influent (approx.)	Sampling time
WWTP1	Västerås	121 000	43 824	18%	24 h
WWTP2	Örebro	140 000	40 200	n.a.	24 h
WWTP3	Eskilstuna	95 000	43 655	n.a.	120 h
WWTP4	Linköping	235 000	40 000	20%	24 h
WWTP5	Uppsala	169 000	46 000	15%	144 h

### Water sample extraction

The extraction of the water samples (2.5 L) was conducted using an automatic solid phase extraction system (SPE-DEX, Horizon Technology, Salem, NH, USA) using HLB extraction disks (Atlantic HLB-H Disks, diameter 47 mm; Horizon Technology, Salem, NH, USA). The disks were conditioned with 280 mL methanol and 420 mL Millipore water, and then the wastewater samples were loaded to the disks at a flow rate of 50 mL min^−1^. After washing the disks twice with 24 mL 5% methanol in Millipore water, the disks were dried under vacuum for 30 min, and eluted 3 times with 25 mL methanol. The samples were then evaporated under a gentle nitrogen stream at 35 °C. Final extracts were in 0.5 mL ethanol. As an operational control blank sample, the same volume of Milli-Q water was concentrated by solid-phase extraction as described above.

The enrichment and dilution of the samples were expressed as relative enrichment factor (REF), calculated as described by Escher *et al*.^12^:

$$REF=enrichment\,facto{r}_{SPE}\ast dilution\,facto{r}_{bioassay}$$

The dilution and enrichment factors are calculated by the following equations:

$$enrichment\,facto{r}_{SPE}=\frac{volume\,water\,}{volume\,extract}$$

$$dilution\,facto{r}_{bioassay}=\frac{volume\,of\,extract\,added\,to\,bioassay}{total\,volume\,of\,bioassay}$$

The initial water value was 2.5 L per sample which was extracted to a volume of 0.5 mL, leading to an enrichment factor_SPE_ of 5000. When incubated with the cells, the concentrated water samples were diluted by 100 fold with the cell medium to get a final concentration of 1% ethanol and a REF of maximum 50. The concentrated water samples were then analyzed in a dilution series with lower REF values. A REF >1 means that the water sample is concentrated, while a REF <1 means that the water samples is diluted as compared to the initial water.

### Bioanalysis

The concentrated water samples were analyzed in quadruplicate in seven bioassays, representing important toxicity endpoints. The applied bioassays are summarized in Table 2 and detailed information on the methods are provided in Supplemental Information (section SI-1). All cells used for this study were from already established cell lines and no animal experiments were conducted in this study. The operational control blank sample was analyzed in all bioassays and found to have no biological activity (data not shown).

**Table 2:** Summary of the applied bioanalytical methods.

Endpoint	Assay	Additional treatment	Positive control	EC/IC value reported	Reference
Androgen receptor activation	AR-EcoScreen	—	DHT	EC_20_	^43^
Androgen receptor antagonism	AR-EcoScreen	DTH	Hydroxy-flutamide	IC_20_	^43^
Estrogen receptor activation	VM7Luc4E2	—	Estradiol	EC_50_	^31^
Estrogen receptor antagonism	VM7Luc4E2	Estradiol	Raloxifen	IC_50_	^31^
Oxidative stress response (Nrf2 activity)	Stably transfected HepG2 cells	—	tBHQ	EC_IR1.5_	
Aryl hydrocarbon receptor activation	Transiently transfected HepG2 cells	—	TCDD	EC_20_	^4, 44^
NFĸB activation	Stably transfected HepG2 cells	—	TNFα	EC_IR1.5_	

### Data evaluation

Bioactivities of water samples and positive controls were normalized to plate vehicle controls, set to 1. For nuclear receptor based assays, standard curves for positive controls were obtained by fitting data to a four parameter sigmoidal curve fit using GraphPad Prism 7. For Nrf2 and NFĸB, the standard curves for positive controls were based in linear regression using GraphPad Prism 7. Water samples were analyzed in dilution series to enable the calculation of effect concentration (EC) values, using linear regression in GraphPad Prism 7. Only data points within the linear range of the concentration-response curve was used for the calculation of EC values, as proposed by Escher *et al*.^26^. For nuclear receptor based assays, the effect concentration 20% or 50% (EC_20_ and EC_50_) were calculated. For Nrf2 and NFĸB activities, the effect concentration for induction ratio 1.5 (EC_IR1.5_) was calculated. The bioanalytical equivalent concentration (BEQ) for REF 1 for each sample was calculated using the linear range of the concentration-response curve and the dose-response relationship for the positive control, corrected for the dilution factor. Removal efficiency was calculated by comparing the BEQ value at REF 1 for WWTP inlet and outlet samples. For each bioassay, the limit of detection (LOD) was calculated as 1 plus 3 times the standard deviation (SD) of the normalized vehicle control values. For antagonistic assays, the LOD was calculated as 1 minus 3 times the SD. The LOD for each assay is presented in the Supplemental Information (Table SI-2).

## Results and Discussion

### Cell viability

Initially, all samples were tested for cytotoxicity at REF 50 and REF 25 in HepG2, AR-EcoScreen and VM7Luc4E2 cells (Figure SI-1). Cytotoxicity was defined as a cell viability of <75% compared to the vehicle treated control. Samples causing cytotoxicity at REF 25 were tested in further dilutions. The main aim of this cell viability testing is to ensure that the bioanalytical assessment of specific parameters is performed under non-cytotoxic conditions.

### Androgen receptor activity

The androgenic activity of each sample was tested in the AREcoScreen assay with DHT as the positive control. Samples were analyzed in dilution series to establish dose-response relationships and calculate EC_20_. Androgenic activity was observed in all samples (Figure SI-2A), with more pronounced activities in the inlet wastewater samples as compared to the outlet water samples. Cytotoxicity was observed (see above and Figure SI-1) only for a few samples at REF 50 and 25 in this cell line, when analyzed with the MTS cell viability test. We could, however, observe concentration-response relationships in the reporter gene assay that could imply cytotoxic effects also at lower REFs. For example, a biphasic concentration-response relationship was observed for multiple samples with increasing concentrations leading to a decrease in the reporter activity at higher REFs (Figure SI-2A). We hypothesize that the observed effect is due to cytotoxicity not detected by the MTS, and conclude that these concentrations should be omitted from the calculation of EC_20_ values.

EC_20_ values (Table 3) were calculated using the data-points in the linear range of the dilution series, at non-cytotoxic concentrations (Figure SI-2B). The inlet wastewater samples for WWTPs 1, 4 and 5 showed an effect clearly above EC_20_ also at the lowest analyzed concentration, and these samples were therefore analyzed at even lower concentrations (Figure SI-2C–D). All inlet wastewater samples exerted androgenic activities at very low REF values, in the range of REF 0.001–0.003. For outlet water samples, EC_20_ values were observed in the range of REF 3.9–5.3. The drastic increase in EC_20_ values for the outlet water samples compared to the inlet wastewater samples indicates that the studied WWTPs had a high removal efficiency for the compounds causing the androgenic activity. Nivala *et al*.^27^ have reported EC_10_ values for androgenic effects of REF 0.5–0.8 for a German WWTP. Välitalo *et al*.^8^ have reported results for the removal efficiency of androgenic compounds in Finnish WWTPs with lowest observed effect concentration (LOEC) increasing from REF 1 for the inlet wastewater water to REF >25 for the outlet water for most studied plants. Similar results have been reported by Leusch *et al*.^28^ who observed high levels of androgenic compounds in untreated wastewater, while all the outlet water samples were below the limit of detection for androgenicity. These two papers report drastic decreases in androgenicity between WWTP inlet and outlet samples which is in line with our results, although we observe effects already at much lower REFs as well as a clear androgenic activity in the outlet water samples. König *et al*.^2^ have reported an EC_10_ value in the range REF 10–20 for a water sample collected a few hundred meters downstream of the effluent discharge from a Serbian WWTP.

**Table 3:** EC values in the units of relative enrichment factor (REF) for all samples in all bioassays.

Assay	WWTP1 Inlet	WWTP1 Outlet	WWTP2 Inlet	WWTP2 Outlet	WWTP3 Inlet	WWTP3 Outlet	WWTP4 Inlet	WWTP4 Outlet	WWTP5 Inlet	WWTP5 Outlet
AR agonism EC_20_ (REF)	0.002	5.3	<0.39	3.9	<0.39	5.2	0.01	3.9	0.03	5.3
AR antagonism IC_20_ (REF)	—	—	—	—	—	—	—	—	—	—
ER agonism EC_50_ (REF)	<0.05	0.8	0.1	1.1	0.1	2.6	<0.05	3.3	0.1	3.1
ER antagonism IC_50_ (REF)	—	—	—	—	—	—	—	—	—	—
Nrf2 activity EC_IR1.5_ (REF)	30	—	10	—	26	—	—	—	8.1	47
AhR activity EC_20_ (REF)	3.4	12.8	5.2	13.9	6.2	23	0.4	8.2	3.4	19
NFĸB activity EC_IR1.5_ (REF)	—	—	1.7	—	—	—	0.3	—	—	—

(–): no effect detected.

The effect at REF 1 was also expressed as DHT equivalent concentration (DHTEQ) (Table 4). The DHTEQ in the inlet wastewater samples varied greatly between 0.6 and 60 nM, while the outlet water samples all had a DHTEQ of around 0.3 nM. The DHTEQ values reported by others also vary greatly; van der Linden *et al*.^29^ report DHTEQ values of 2.6–2.8 pM in WWTP outlet samples, Bain *et al*.^30^ observed DHTEQs in the range of 100 pM to 1 nM in the inlet wastewater sample to a WWTP but no androgenic activity in the outlet water, and Välitalo *et al*.^8^ report DHTEQs between 48 and 230 pM in the inlet wastewater to five Finnish WWTPs, but no androgenic activity in the outlet water. When comparing the results it should, however, be noted that these studies have been carried out with a different bioassay for androgenicity, the AR-CALUX system, than our study. Both the AR-CALUX system and the AR-EcoScreen are based on transfected mammalian cells and show a very similar responsiveness to the positive control DHT with EC_50_ values of 0.31 to 0.45 nM for the AR-CALUX^30^ and 0.45 nM for the AR-EcoScreen (Table SI-3).

**Table 4:** Bioanalytical equivalent concentrations (BEQ).

Assay	WWTP1 Inlet	WWTP1 Outlet	WWTP2 Inlet	WWTP2 Outlet	WWTP3 Inlet	WWTP3 Outlet	WWTP4 Inlet	WWTP4 Outlet	WWTP5 Inlet	WWTP5 Outlet
AR agonism DHTEQ (nM)	60	0.2	0.6	0.3	0.7	0.3	40	0.25	38	n.d.
ER agonism E2EQ (pM)	930	30	130	30	100	10	930	4.0	130	4.0
Nrf2 activity tBHQEQ (µM)	2.5	1.1	n.d.	0.8	n.d.	n.d.	3.5	1.0	1.2	0.9
AhR activity TCDDEQ (pM)	580	490	730	350	610	300	1200	480	750	300
NFĸB activity TNFαEQ (ng mL^−1^)	n.d.	n.d.	0.05	n.d.	n.d.	n.d.	0.2	n.d.	n.d.	n.d.

For samples denoted with not detected (n.d.), the calculated response at REF1 was lower than the range of the standard curve.

The removal efficiency, calculated based on the DHTEQ values at REF 1 (Table 5), for androgenic compounds was very high (>99%) for the WWTPs where a high level of androgenic compounds was observed in the inlet wastewater (WWTPs 1 and 4). For the WWTPs where relatively low levels of androgenic compounds was observed in the inlet wastewater (WWTPs 2 and 3), the removal efficiency was lower (50–60%), but the outlet water had DHTEQ values in the same range as the plants with high removal efficiency. Although these five WWTPs have very similar treatment techniques, the removal-efficiency for androgenic compounds varies greatly. The plants with very high removal-efficiency also has a higher DHTEQ level as compared to the plants with lover removal-efficiency and it could be speculated that this difference is due to the mixture of androgenic compounds present in the inlet waters (*e.g*. that the higher level of androgens could compose of compounds that are more easily to remove by the current treatment techniques).

**Table 5:** Removal-efficiencies bioassays where bioactivity was observed in both inlet and outlet water.

Assay	WWTP1	WWTP2	WWTP3	WWTP4	WWTP5
AR agonism	>99%	50%	57%	>99%	n.d.
ER agonism	97%	77%	90%	>99%	97%
Nrf2 activity	56%	n.d.	n.d.	n.d.	25%
AhR activity	16%	52%	51%	60%	60%

Antiandrogenic effects were assayed in the AREcoScreen assay which was stimulated by DHT, with hydroxyflutamide (OHF) used as a positive control for antiandrogenicity. Only concentrations that were non-cytotoxic in both the MTS test and in the AREcoScreen agonism test (discussed above) were included in this assay. One sample showed an antiandrogenic reponse just above the LOD (Figure SI-2E), while all other samples showed no antiandrogenic response at non-cytotoxic concentrations. Due to interference by cytotoxicity, we were unable to calculate an IC_20_ value for this effect. Nivala *et al*.^27^ have also reported that cytotoxicity is masking any antiandrogenic effects in WWTP inlet and outlet samples.

### Estrogen receptor activity

The estrogenic activity of each sample was tested in the VM7Luc4E2 assay^31^ with estradiol as the positive control. Samples were analyzed in dilution series to establish dose-response relationships and calculate effect concentration (EC) values. As expected, estrogen receptor activity was observed in all samples (Figure SI-3A), with more pronounced activities in the inlet wastewater samples as compared to the outlet water samples. Similar to the androgen receptor activity assay, most samples showed a biphasic concentration-response relationship with decreasing reporter activity with increasing concentration at high REF values, even where the cell viability test did not indicate cytotoxicity. We hypothesize that this effect is caused by cytotoxicity not detected by the luminescent ATPase cell viability test, and excluded these high REF values from the analysis.

All inlet wastewater samples reached the maximum assay response already at very low REF values, and were therefore reanalyzed in further dilutions to enable the calculation of EC values (Figure SI-3C). As most samples had EC_20_ values below the range of concentrations analyzed, we report the EC_50_ values for this endpoint. EC_50_ values (Table 3) were calculated at non-cytotoxic concentrations within the linear range of the concentration-response curve (Figure SI-3B and D). The inlet wastewater samples had EC_50_ values in the range of REF <0.05 to REF 0.12 and the outlet water samples in the range REF 0.75 to REF 3.31. All studied plants had a strong increase in EC_50_ between WWTP inlet and outlet samples, indicating an efficient removal of estrogenic compounds during the water treatment process.

In similarity to our findings, Välitalo *et al*.^8^ have reported estrogenic activities in both inlet and outlet waters from WWTPs, analyzed by a mammalian reporter gene assay similar to the one used in this study. Välitalo *et al*. also report that the estrogenicity was markedly decreased in the outlet water samples as compared to the inlet wastewater samples. Chou *et al*.^32^ have investigated the removal efficiency of estrogenic compounds during wastewater treatment in a Taiwanese WWTP and reported strongly decreasing ER activity in the outlet water samples as compared to the inlet wastewater sample. The findings in both these studies are in concordance with the findings in the current study. Escher *et al*.^12^ have reported EC_10_ values in the range of REF 0.07–4.2 for the effluent water from two different WWTPs, but the study does not include any analysis of the inlet wastewater to those plants. Nivala *et al*.^27^ have reported EC_10_ values of REF 0.1–0.3 for the inlet wastewater and REF 3.8–6.4 for the outlet water in a German WWTP. Most of these studies have analyzed samples from WWTPs with primary, secondary, and tertiary treatment steps, but the study by Chou *et al*.^32^ have studied samples from a thin film transistor liquid crystal display WWTP and Escher *et al*.^12^ report results for the secondary treated sewage effluent water which serves the as influent to water reclamation plants. At this stage, due to the limited number of studies, it is not possible to conclude that any of the studied wastewater treatment techniques is superior for removal of estrogenic compounds. In addition to difference in the treatment techniques, it can be hypothesized that the mixture of estrogens are varying greatly between the different study sites, also influencing the removal-efficiency.

A study by König *et al*.^2^ have examined the estrogenicity in water samples collected upstream and downstream of a WWTP effluent discharge in Serbian River Danube. Upstream of the effluent discharge, the EC_10_ value for ER activity was in the range of REF 5–100 depending on the assay used for detecting the effect. A few hundred meters downstream of the effluent discharge, the EC_10_ was found to be in the range of REF 0.1–2, indicating that the discharged WWTP effluent water contains estrogenic compounds. The same was found for the Ammer River, Germany, where the EC_10_ was REF 37.9 upstream of a large scale WWTP and REF 3.1 downstream of the plant.

The estradiol equivalent concentration (E2EQ) was calculated at REF 1 (Table 4). The inlet wastewater samples had an E2EQ in the range of 100–930 pM while the outlet water samples had an E2EQ in the range 4–30 pM. The removal efficiency for estrogenic compounds was >75% for all WWTPs and ≥90% for four out of five studied WWTPs (Table 5). The E2EQ values observed in this study are comparable with many other reports; Miege *et al*.^33^ report E2EQ of 11 pM in the outlet water from a WWTP, Murk *et al*.^34^ observed E2EQs of 3–440 pM in WWTP inlet samples and 0.1–58 pM in outlet water samples, Nivala *et al*. have recently reported E2EQs in the range of 36–88 pM in WWTP inlet waters and 1.4–2.5 pM in the outlet water, Välitalo *et al*.^8^ calculated the E2EQs to 55–147 pM in the inlet wastewater of five Finnish WWTPs and <18 pM in the outlet water from all plants, and Bain *et al*.^30^ observed E2EQ values of 110–440 pM in the inlet wastewater of three Australian WWTPs and 3–22 pM in the outlet water from the same plants.

Antiestrogenic effects were assayed in the VM7Luc4E2 assay^31^ which was stimulated by estradiol with raloxifen used as a positive control for antiestrogenicity. Only concentrations that were non-cytotoxic in both the luminescent ATPase cell viability test and in the VM7Luc4E2 agonism test were included in this assay. Antiestrogenic response were not observed in any inlet wastewater sample (Figure SI-3E). For most outlet water samples (WWTPs 2, 3, 4 and 5) we observed ER activities below the LOD for the antiestrogenic response, but the effects were ambiguous and with no clear concentration-response relationships.

### Oxidative stress response (Nrf2 activity)

Oxidative stress response was assayed as the activity of Nrf2 in stably transfected HepG2 cells. Most inlet wastewater samples activated the Nrf2 pathway at high REF values (Figure SI-4), with EC_IR1.5_ values in the range REF 8.1–30. No Nrf2 activity was detected for four out of five outlet water samples (Figure SI-4). In the outlet water sample from WWTP5, Nrf2 activity was observed with EC_IR1.5_ of REF 47, as compared to 8.1 for the inlet wastewater. These results indicate that the treatment processes in the studied WWTPs efficiently removes oxidative stress inducing compounds from the water, as shown by the increasing EC_IR1.5_ values between WWTP inlet and outlet samples for each plant.

The Nr2 activities observed in this study are very low compared to other studies of wastewater. König *et al*.^2^ have reported an Nrf2 activity with EC_IR1.5_ of REF <10 for a sample collected in a river a few hundred meters downstream of a WWTP effluent discharge, Echer *et al*.^12^ have reported Nrf2 activities with EC_IR1.5_ of REF 1–10 in effluent water from Australian WWTPs, Müller *et al*.^35^ report an EC_IR1.5_ of REF 5.5 for a water sample collected in the German Ammer River, downstream of a large scale WWTP, and Nivala *et al*.^27^ have reported EC_IR1.5_ values of around REF 0.3 for the inlet wastewater in a German WWTP and REF 1.5–1.9 for the outlet water.

### Aryl hydrocarbon receptor activity

The AhR activity was assayed in HepG2 cells transiently transfected with a luciferase plasmid responsive to ligand-activated AhR and a renilla plasmid to control for transfection efficiency. Generally, we observed relatively high AhR activities in the inlet wastewater for all five WWTPs with EC_20_ values in the range REF 0.44–6.2 (Table 3 and Figure SI-5A and B). For all treatment facilities we observed a clear increase in the EC_20_ in the outlet water as compared to the inlet wastewater, indicating that all treatment facilities removes AhR inducing chemicals from the water. For the outlet water samples, EC_20_ values were in the range of REF 8.2–23.

For two WWTPs (WWTP 1 and WWTP 4), the inlet wastewater sample showed a clear decrease in the renilla luciferase activity at the highest REF values, although the general cell viability test did not indicate any problem with cytotoxicity. A strong decrease in the renilla luciferase activity can cause false positive results in bioassays based on transiently transfected cells. Therefore, water samples for the inlet wastewater for WWTP 1 and WWTP 4 were only analyzed at lower REF values.

The EC values observed in this study are similar to other reports of AhR activities in wastewater. An Australian study including two WWTP effluent samples reported EC_10_ values for the AhR activity of REF 1,2. For the German Ammer River, Müller *et al*.^35^ report an EC_10_ value of REF 8.4 upstream of a WWTP effluent discharge and REF 2.0 downstream of the plant. Other studies have reported conflicting results. Long *et al*.^36^ have reported decreased levels of AhR active compounds in the outlet water from WWTPs as compared to the inlet wastewater in two Danish WWTPs, while Chou *et al*.^32^ report unsatisfactory removal of AhR agonists during wastewater treatment in a Taiwanese WWTP. Nivala *et al*.^27^ report that the EC_10_ value for AhR activation was REF 0.7 in the inlet wastewater to a German WWTP and REF 1.5–1.6 in the outlet water.

The TCDD equivalent concentration (TCDDEQ) values were calculated for REF 1, and found to vary between 580 and 1200 pM for the inlet wastewater samples and 300–480 pM for the outlet water samples (Table 4). The observed TCDDEQs are high compared to other studies, for example Nivala *et al*.^27^ that report TCDDEQ values of around 0.8 pM in the inlet wastewater to a German WWTP and around 0.4 pM in the outlet water.

The removal efficiency for AhR activating compounds was 50–60% for most WWTPs studied, but WWPT 1 had a removal efficiency as low as 16% (Table 5).

### NFĸB activation

The NFĸB activity was assayed in HepG2 cells stably transfected with an NFĸB responsive luciferase plasmid. For most samples, no NFĸB activity was observed (Figure SI-6A). For some samples (outlet water from WWTP 2 and both samples from WWTP 3), we observed NFĸB activity above the threshold for induction of induction ratio 1.5, but with no clear concentration-response relationship (Figure SI-6A). For two inlet wastewater samples (WWTP 2 and 4), we observed NFĸB activity above the threshold for induction of induction ration 1.5. The linear range of the concentration-response relationship was used to calculate the EC_IR1.5_ value, which was found to be REF 1.7 for WWTP 2 and REF 0.33 for WWTP 4 (Figure SI-6B). The observed EC_IR1.5_ values for these two samples are similar to a previously published report studying NFĸB activity in WWTP inlet and outlet waters^27^. The TNFα equivalent concentration (TNFαEQ) for these two samples were calculated to be 0.05–0.2 ng mL^−1^ (Table 4).

NFĸB activity in water samples can be caused by either environmental pollutants or by the presence of endotoxins in the water. It has recently been demonstrated that bacterial lipopolysaccharides (LPS), and potentially other endotoxins, can be co-extracted from the water to the extract when using solid phase extraction methods^37^. In that study, Neale *et al*.^37^ also report that the majority of the observed NFĸB effect in four Australian environmental water samples was caused by endotoxins and not environmental pollutants.

### Comparing observed activities with effect-based trigger values

To enable an evaluation of the hazards and risks associated with observed bioactivities in studies using *in vitro* methods, effect-based trigger values (EBTs) are currently being developed. The EBT is a threshold level for the bioactivity, and if a sample shows higher activity than the EBT it causes concern for the endpoint the EBT is developed for. There is currently not a scientific consensus regarding which actual EBT values that should be used, and the EBT value is also dependent on the assay used in the study, the positive control used to calculate the BEQ, and which endpoint the value is developed for (*e.g*. human health endpoints or ecotoxicological endpoints). As an example, Escher *et al*.^38^ have proposed EBTs for a number of *in vitro* bioassays, based on a read-across approach from the guideline values in the Australian Guidelines for Water Recycling: Augmentation of Drinking Water Supplies. Of relevance for the *in vitro* bioassays used in this study, the authors proposed EBT values of 14 ng testosterone/L for androgenic effects, 0.2–1.8 ng estradiol/L (0.7–6.6 pM) for estrogenic effects. It is not possible to compare the proposed EBT value for androgenic activity or AhR activity with the activities observed in this study, as we have used a different reference compound. For estrogenic activity, we observed E2EQ values in the range 0.04–0.3 pM for the effluent waters, which is clearly below the proposed EBT values. It should, however, be noted that these EBT values have been developed with *in vitro* bioassays that are similar but not identical to the one used in this study. In a different study, van der Oost^39^ has proposed EBT values derived from ecotoxicological endpoints. For estrogenic activities, the authors propose an EBT value of 0.5 ng estradiol/L (1.8 pM), which is clearly above the effects that we observe in the effluent water. For AhR activities, van der Oost propose an EBT of 50 pg TCDD/L (0.15 pM) based on observed effects of TCDD on the reproduction of rare minnow (*Gobiocypris rarus*) after exposure to TCDD. The TCDDEQs observed in this study is clearly above the proposed EBT which causes concern and prompts further research regarding which compounds that causes the effect and how these can be removed during wastewater treatment.

### Using bioanalytical tools to evaluate toxicity removal efficiency in wastewater treatment techniques

WWTPs are challenged by the increasing risk of chemical pollutants, of which many are unknown, in the inlet wastewater^1,2,8,40^. Many WWTP operators are currently planning investments in new treatment technologies to target the chemical pollutants during the treatment process and are therefore in need of methodologies to evaluate the removal efficiency of bioactive chemicals both in the current treatment process and in potential new treatment techniques^9,25,27^, to make sound financial investments. Bioanalytical tools shows great promise to perform such evaluations, since they make it possible to evaluate the effects of all bioactive compounds in a water sample, without any *a priori* information on the chemical identity of the causative compounds. Aquatic effect-based monitoring tools, such as bioassays, has also been proposed as a part of the revised European Water Framework Directive^41^. In this study, we have demonstrated that *in vitro* bioassays based on mammalian cells, assaying initiating molecular events in key toxicity pathways, can be used for effect-based monitoring of wastewater treatment facilities and in our opinion these methods can be valuable both for monitoring purposes and to evaluate the effectiveness of new treatment techniques.

## Conclusions

**Figure 1:**
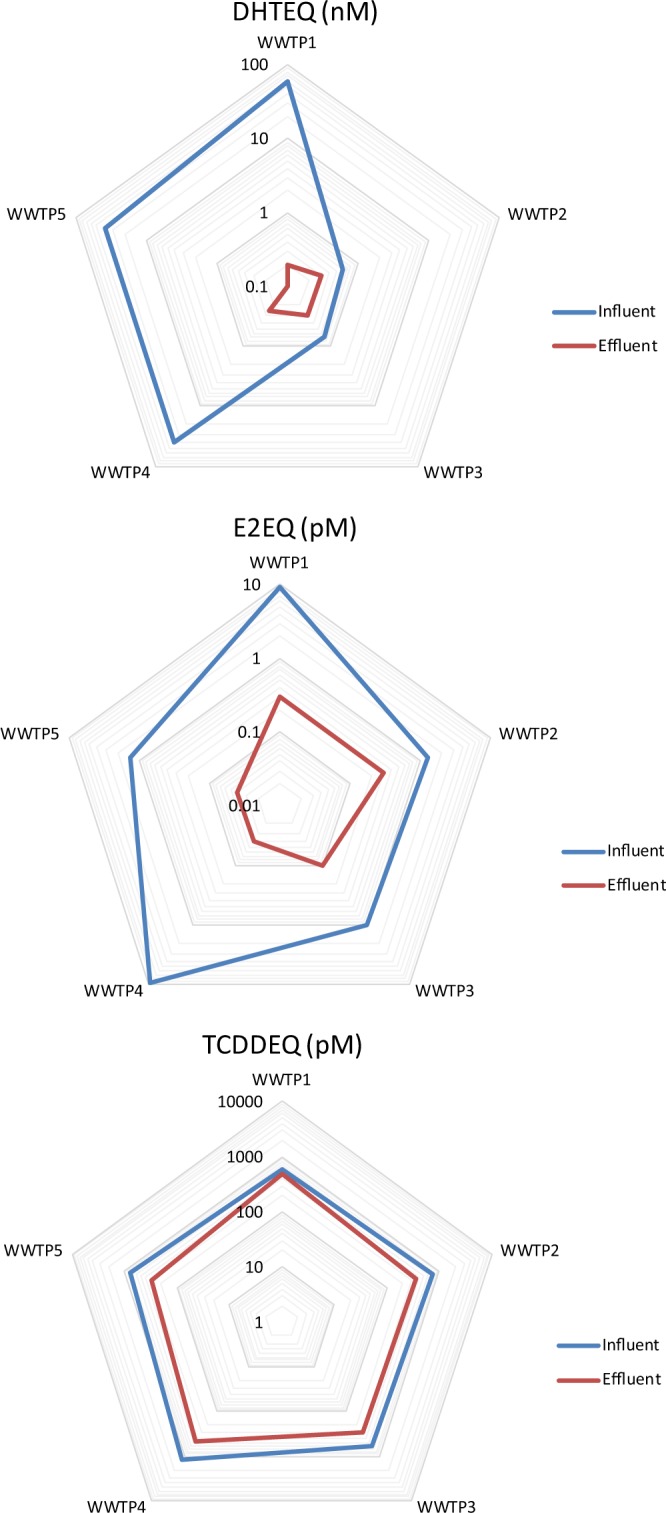
Radar plots showing bioanalytical equivalent concentrations (DHTEQ for AR activity, E2EQ for ER activity, and TCDDEQ for AhR activity) for influent and effluent water for the five studied WWTPs.

In this study, we have demonstrated that *in vitro* bioanalytical tools can be used to evaluate the removal efficiency of bioactive chemical pollutants during wastewater treatment. The great strength with this methodology, over classical target chemical analysis, is that it can measure the biological effects of both known and unknown chemical pollutants. For the nuclear receptor mediated effects (ER, AR, AhR) we observed bioactivity in all inlet samples, but generally the removal efficiency for these compounds was high during the water treatment process. The bioanalytical equivalent concentration for these three end-points (E2EQ, DHTEQ and TCDDEQ, respectively) for inlet and outlet samples (Fig. 1) highlights the high removal efficiency, especially for compounds activating ER and AR. Adaptive stress responses such as Nrf2 and NFĸB activites was, with one exception, only found in the inlet wastewater to the WWTPs. This study lends support to the previous suggestions^4,8,27,30,41,42^ that *in vitro* bioassays can be valuable tools to evaluate the effectiveness in wastewater treatment processes, and more general to monitor the presence of bioactive chemical pollutants in the aquatic environment. Further development is, however, needed regarding bioassays to include, standardized testing protocols, and consensus EBT values.

## Supplementary information

Supplementary information

## Data Availability

The datasets generated during the current study are available from the corresponding author upon request.
