# Supplementary material for: In vitro bioanalytical evaluation of removal efficiency for bioactive chemicals in Swedish wastewater treatment plants
Source: Sci Rep. 2019 May 9;9:7166. doi: 10.1038/s41598-019-43671-z (PMC6509133; doi:10.1038/s41598-019-43671-z)
Supplement: Supplementary file 1 — Supplementary information [file 41598_2019_43671_MOESM1_ESM.pdf]

Supplementary information:

In vitro bioanalytical evaluation of removal efficiency for bioactive chemicals in Swedish wastewater treatment plants

Johan Lundqvist[a*], Geeta Mandava[a], Sebastian Lungu-Mitea[a], Foon Yin Lai[b], Lutz Ahrens[b]

[a]Department of Biomedical Sciences and Veterinary Public Health, Swedish University of Agricultural Sciences, Box 7028, SE-750 07 Uppsala, Sweden

[b]Department of Aquatic Sciences and Assessment, Swedish University of Agricultural Sciences, Box 7050, SE-750 07 Uppsala, Sweden

*Corresponding author:
Johan Lundqvist
Department of Biomedical Sciences and Veterinary Public Health, Swedish University of Agricultural Sciences
E-mail: johan.lundqvist@slu.se

Section SI-1: Detailed information on the bioanalytical methods

Table SI-1: Additional information regarding WWTPs

Table SI-2: Limit of detection (LOD) for the different bioassays

Table SI-3: Effect concentration values for positive controls

Figure SI-1: Cell viability of HepG2, AREcoScreen, and VM7Luc4E2 cells

Figure SI-2 – SI-6: Results for bioanalytical testing of water samples.

Section SI-1: Detailed information on the bioanalytical methods

Cell culture

The human hepatocellular carcinoma (HepG2) cell line was used for aryl hydrocarbon receptor (AhR) reporter gene assays. Additionally, stably transfected HepG2 cell-lines were used for the Nrf2 and NFκB reporter gene assay (Signosis Inc., Santa Clara, CA, USA). Non-transfected HepG2 and Nrf2 stably transfected cells were cultured in Dulbecco's Modified Eagle Medium (with 4.5 g/L glucose) (DMEM) (Lonza, Basel, Switzerland) supplemented with 2 mM L-glutamine (Lonza, Basel, Switzerland), an antibiotic-antimycotic solution with a final concentration of 100 U mL^{-1} penicillin, 100 µg mL^{-1} streptomycin, 0.25 µg mL^{-1} amphotericin B) (Gibco, Thermo Fisher Scientific), and 10% fetal bovine serum (FBS) (Gibco, Thermo Fisher Scientific). NFκB stably transfected HepG2 cells were cultured in DMEM (with 4.5 g L^{-1} glucose) (Lonza, Basel, Switzerland) supplemented with 10% FBS (Gibco, Thermo fisher Scientific), Penicillin/Streptomycin with a final concentration of 100 U mL^{-1} penicillin, 100 µg mL^{-1} streptomycin, (Lonza). Culture medium for stably transfected Nrf2 and NFκB cells further had 100 µg mL^{-1} of Hygromycin B (InvivoGen, USA).

The Chinese Hamster Ovary (CHO) cell line (AR-EcoScreen, purchased from National Institutes of Biomedical Innovation, Health, and Nutrition JCRB cell bank) was used for the AR reporter gene assay. These stably transfected CHO cells were cultured in DMEM F12 (Sigma) medium supplemented with 5% FBS (Gibco), Penicillin/Streptomycin with a final concentration of 100 U mL^{-1} penicillin, 100 µg mL^{-1} streptomycin, (Lonza) 2 mM L-glutamine (Lonza, Basel, Switzerland), 100 µg mL^{-1} Hygromycin B (InvivoGen, USA), and 200 µg mL^{-1} Zeocin (Invitrogen, CA, USA). Experimental medium consists of DMEM F12 (Sigma) medium supplemented with 5% dextran-charcoal treated fetal bovine serum (Thermo Scientific), 4 mM L-glutamine (Gibco, Thermofisher Scientific), Penicillin/Streptomycin with a final concentration of 100 U mL^{-1} penicillin, 100 µg mL^{-1} streptomycin, (Lonza).

VM7Luc4E2 cells, stably transfected to assay ER activity, were routinely cultured in RPMI 1640 (Gibco) supplemented with 8% fetal bovine serum (FBS) (Gibco) and 0.9% penicillin-streptomycin (100 U mL^{-1} penicillin, 100 µg mL^{-1} streptomycin, Lonza). 0.55 mg/ml Gentamicin (Gentamicin Sulfate, 50mg mL^{-1}, Lonza) was used as positive selector. VM7Luc4E2 cells, were grown in experimental medium consisting of DMEM medium (with 4.5 g L^{-1} glucose) (Lonza), 4.5% dextran-charcoal treated fetal bovine serum (Thermo Scientific), 2% L-glutamine (Lonza,

Basel, Switzerland), 0.9% penicillin-streptomycin (100 U mL^{-1} penicillin, 100 µg mL^{-1} streptomycin, Lonza) and 0.38 mg mL^{-1} Gentamicin (Gentamicin Sulfate, 50 mg mL^{-1}, Lonza) was used as positive selector.

The cells were cultured in incubator with humidified atmosphere at 37˚C containing 95% air and 5% CO_2. Medium was changed every 2-3 days. Trypsin-EDTA (Gibco, Thermofisher Scientific) was used for sub-culturing of cells.

Cell treatment

All concentrated waste water samples were tested in cell viability assay and reporter gene assays in quadruplicates. In all experiments, vehicle control was included, consisting of 1% ethanol, equivalent to the ethanol concentration in the exposure groups. Vehicle controls were tested in eight replicates.

Cell viability assessment

The MTS-based colorimetric assay (Cell Titer 96® Aqueous One Solution Cell Proliferation Assay) (Promega) was used for HepG2 and CHO cells to assess the cell viability. HepG2 cells were seeded, $2x10^4$ cells $well^{-1}$, in transparent 96-well plates (Costar® Corning Incorporated), and left to incubate for 48 hours, after which medium was changed. After another 24 h, cells were treated with waste water samples for 24 hours. CHO cells were seeded 96-well transparent plates, $9x10^3$ cells $well^{-1}$, and left to incubate for 24 hours, after which cells were exposed to waste water samples for 24 hours. At experiment termination, cell viability assay reagent, 20 µL $well^{-1}$, was added and cells were incubated for approximately 15 minutes (HepG2) and 30 minutes (CHO).

CellTiter-Glo® Luminescent Cell Viability Assay was used for VM7Luc4E2 cells. Cells were seeded, $4x10^4$ cells $well^{-1}$, in white clear bottomed 96-well plates (Corning, USA) and incubated for 24 hours after which cells were exposed to waste water samples for 24 hours. At assay termination, 50 µL $well^{-1}$ of reagent was added, shaken for 2 minutes, and successively incubated for 10 minutes.

Absorbance and luminescence for the MTS and ATP-based assay, respectively, was measured on a Wallac Victor 1420 microplate reader (PerkinElmer, Waltham, MA, USA). In case of observed compromised cell viability, samples were tested in dilutions to determine the lowest non-cytotoxic concentration to be included for further testing in reporter gene assays.

Reporter gene assays

The AhR reporter gene assay was based on transient transfection protocol in HepG2 cells, whereas the remaining reporter gene assays, were based on stably transfected cell lines. Positive standards were included in all experiments.

HepG2 cells were seeded in white clear bottomed 96-well plates (Corning, USA), $2x10^4$ cells $well^{-1}$ and left to incubate for 48 hours after which medium was changed and transfections were performed. Lipofectamine 2000 (0.3 µL $well^{-1}$) (Invitrogen, Thermo Fisher Scientific, USA) was used as transfection reagent. Cells for the AhR reporter gene assay was transfected with a pGL3 based luciferase reporter plasmid (pGudLuc7.5, 90 ng $well^{-1}$) (donated by Professor Michael Denison, University of California, USA) and 30 ng $well^{-1}$ Renilla luciferase plasmid. The transfection mixture was prepared by adding Lipofectamine to 5 µL $well^{-1}$ Opti-MEM (1x) reduced serum medium (Gibco, Thermo Fisher Scientific, USA). After five minutes, the plasmids were added to 5 µL $well^{-1}$ Opti-MEM (1x) reduced serum medium and the two solutions were mixed gently. 10 µL transfection mixture was added to each well on the plate and left for 24 hours after which cells were treated with waste water samples and standards for 24 hours. TCDD (SUPELCO, Sigma-Aldrich, USA) was tested in concentrations of 10-5000 pM in the AhR reporter gene assay. At assay termination, cells were lysed with passive lysis buffer (20 µL $well^{-1}$) (Promega, USA) for 15 minutes. White adhesive sealing film was attached to plate bottom for the AhR reporter gene assay and total lysate was used for activity measurement. The Dual-Luciferase® Reporter Assay System (Promega, USA) was used to measure Renilla and Firefly luciferase activity according to manufacturer's protocol. Luminescence was measured on a Tecan plate reader (TECAN, Austria GmbH, Austria) with two automatic injection syringes. The injection volume for the Firefly luciferase reagent and Renilla luciferase reagent was 45 µL $well^{-1}$. Luminescence measurement was conducted over a 5 s period, 2 s after reagent automatic injection with Firefly and then Renilla luciferase reagent addition.

All experiments for stably transfected cell lines were conducted in white clear-bottomed 384 well plates (Corning, NY, USA) over a three day period with cell seeding on day 1, cell treatment with concentrated waste water samples and standards on day 2, and luciferase measurement on day 3. Nrf2 and NFkB stably transfected HepG2 cells were seeded, 6800 cells $well^{-1}$ and 4000 cells $well^{-1}$ respectively, in culture medium without Hygromycin B. tert-

Butylhydroquinone (tBHQ) (Sigma Aldrich, USA) was used as a standard in the Nrf2 reporter gene assay and tested in concentrations of 0.78-25 µM, and TNFα was used for NFκB reporter gene assay ranging from 0.2-50 ng mL^{-1}.

VM7Luc4E2 cells were seeded, $1.6 x 10^4$ cell $well^{-1}$, in experimental medium and 4000 cells $well^{-1}$ of CHO cells in experimental medium. The ER and AR reporter gene assay was conducted in agonist and antagonist mode. 17β-estradiol (E2) (Sigma-Aldrich, USA) was used as a standard in the ER reporter gene assay and tested in concentrations in the range of $3.67 x 10^{-10}$ M to $3.59 x 10^{-13}$ M. In the ER antagonistic mode, $9.18 x 10^{-11}$ M of E2 was added together with the waste water samples and standards to stimulate the ER and raloxifen was used as a positive control in concentrations from $2.45 x 10^{-8}$ M to $9.57 x 10^{-11}$ M. In the AR antagonistic mode, 500 pM DHT was added together with the waste water samples and standards to stimulate the AR. In the AR reporter gene assay, the positive control for activation of the receptor was dihydrotestosterone (DHT) (Sigma-Aldrich, USA) tested in concentrations in the range of 10^{-6} M to 10^{-12} M, and in the AR antagonism mode the positive control was hydroxyflutamide (OHF) (Sigma-Aldrich, USA) tested in concentrations in the range of 10^{-5} M to 10^{-10} M.

At experiment termination, cells for reporter gene assay were lysed with passive lysis buffer (PLB) (Promega), 10 µL $well^{-1}$, for 15 minutes in 384 well plates. Luciferase activity was measured using the Luciferase® Reporter Assay System (Promega) according to the manufacturer's instructions. Luminescence was measured on a Tecan plate reader (TECAN, Austria GmbH, Austria) with an automatic injection syringe. The injection volume for the Firefly luciferase reagent was 10 µL $well^{-1}$. Luminescence measurement was conducted over a 5 s period, 2 s after reagent was automatically injected with Firefly luciferase reagent. White adhesive sealing film was attached to plate bottom before measurement.

Table SI-1: Additional information regarding WWTPs, annual mean values according to the latest available environmental report for each plant.

	P_{tot} (mg/L)	N_{tot} (mg/L)	NH4-N effluent (mg/L)	Suspended solids effluent (mg/L)	Recipient type
WWTP1	5.1 (influent)<br>0.2 (effluent)	37 (influent)<br>12 (effluent)	4.1	2.6	Lake
WWTP2	4.8 (influent)<br>0.2 (effluent)	36 (influent)<br>21 (effluent)	14	7.1	River
WWTP3	5.1 (influent)<br>0.2 (effluent)	14 (influent)<br>14 (effluent)	1.5	n.d.	River
WWTP4	6.2 (influent)<br>0.2 (effluent)	58 (influent)<br>8.4 (effluent)	2.0	<5.8	River
WWTP5	7.0 (influent)<br>0.1 (effluent)	58 (influent)<br>9.7 (effluent)	0.5	<5.3	River

Table SI-2: Limit of detection (LOD) for the different bioassays

**Endpoint**	**Assay**	LOD
Androgen receptor agonism	AR-EcoScreen	1.6
Androgen receptor antagonism	AR-EcoScreen	0.7
Estrogen receptor agonism	VM7Luc4E2	1.3
Estrogen receptor antagonism	VM7Luc4E2	0.7
Oxidative stress response (Nrf2 activity)	Stably transfected HepG2 cells	1.9
AhR activation	Transiently transfected HepG2 cells	2.1
NFκB activation	Stably transfected HepG2 cells	1.6

Table SI-3: Effect concentration values for positive controls

**Assay**	Value reported	EC value
AR agonism	EC_{50}	0.45 nM
AR antagonism	IC_{50}	0.03 µM
ER agonism	EC_{50}	7 pM
ER antagonism	IC_{50}	0.01 nM
Nrf2 activity	$EC_{IR1.5}$	4 µM
AhR activity	EC_{20}	912 pM
NFκB activation	$EC_{IR1.5}$	0.09 ng mL^{-1}

Figure SI-1

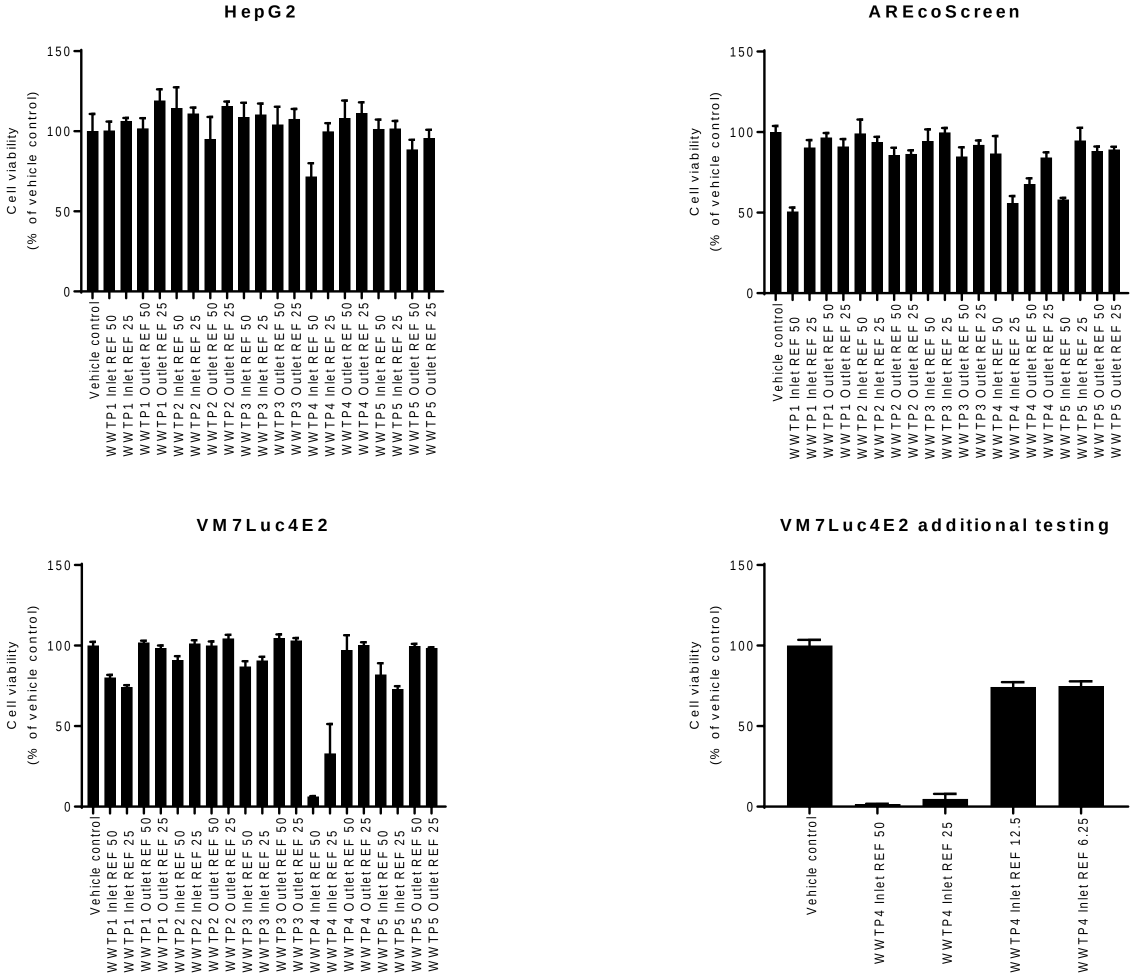

Figure SI-1. Cell viability of HepG2, AREcoScreen, and VM7Luc4E2 cells following 24 h exposure to concentrated water samples (n=4). Cell viability was normalized to vehicle control, set to 100%.

Figure SI-2A

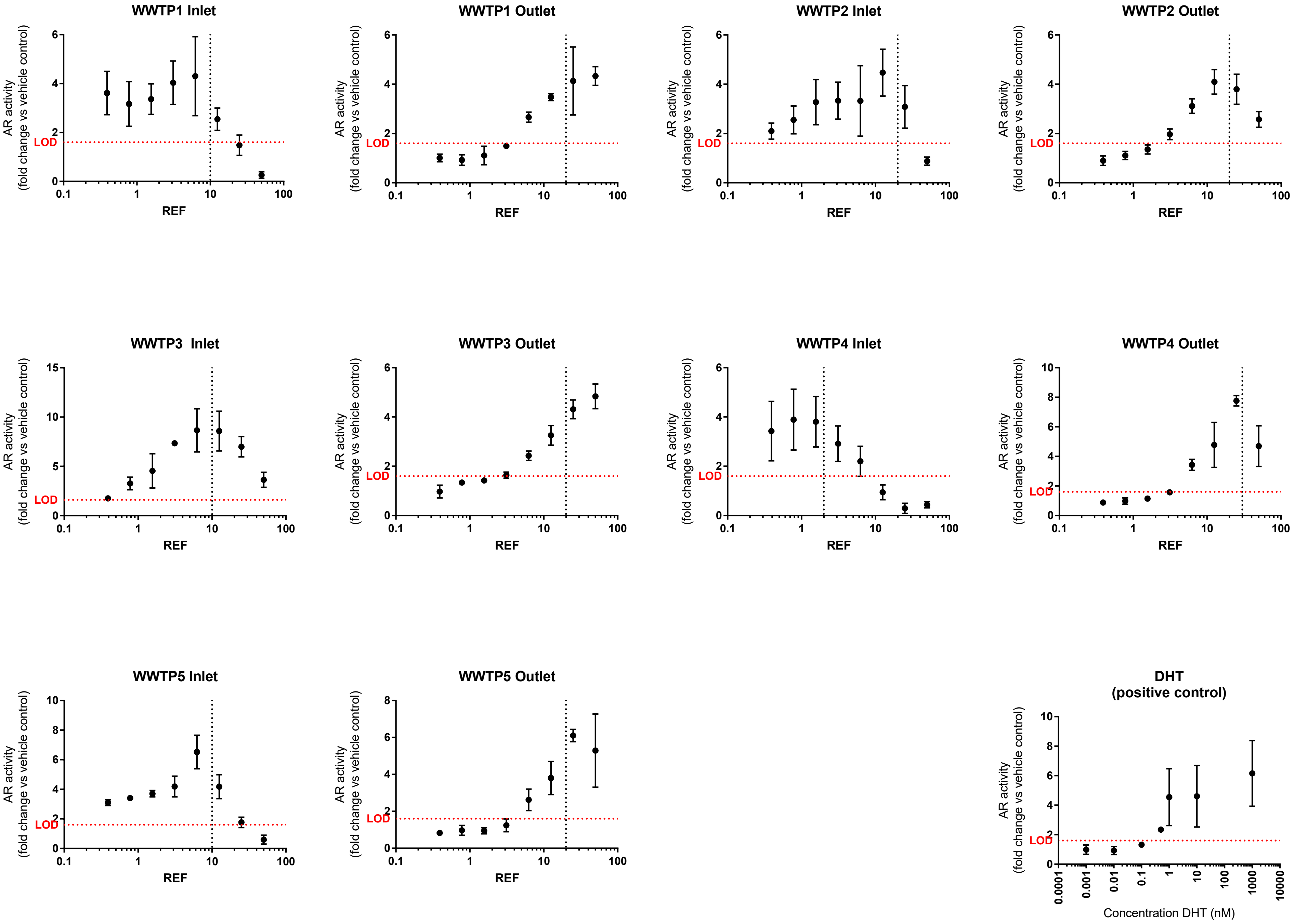
WWTP1 Inlet
WWTP1 Outlet
WWTP2 Inlet
WWTP2 Outlet
WWTP3 Inlet
WWTP3 Outlet
WWTP4 Inlet
WWTP4 Outlet
WWTP5 Inlet
WWTP5 Outlet
DHT
(positive control)
AR activity
(fold change vs vehicle control)
REF
LOD
Concentration DHT (nM)

Figure SI-2B

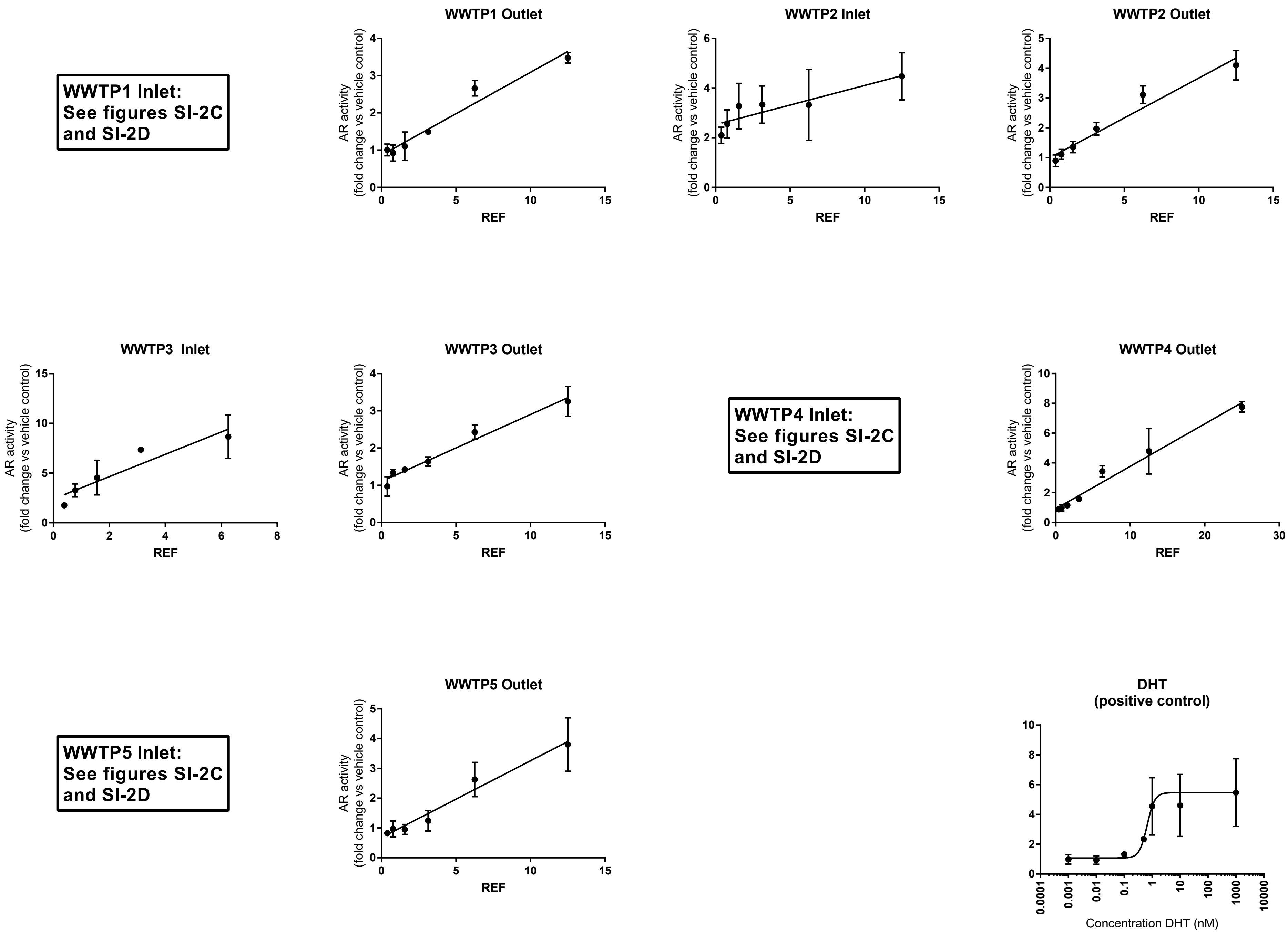
WWTP1 Inlet:
See figures SI-2C
and SI-2D
WWTP1 Outlet
WWTP2 Inlet
WWTP2 Outlet
WWTP3 Inlet
WWTP3 Outlet
WWTP4 Inlet:
See figures SI-2C
and SI-2D
WWTP4 Outlet
WWTP5 Inlet:
See figures SI-2C
and SI-2D
WWTP5 Outlet
DHT
(positive control)
AR activity
(fold change vs vehicle control)
REF
Concentration DHT (nM)

Figure SI-2C

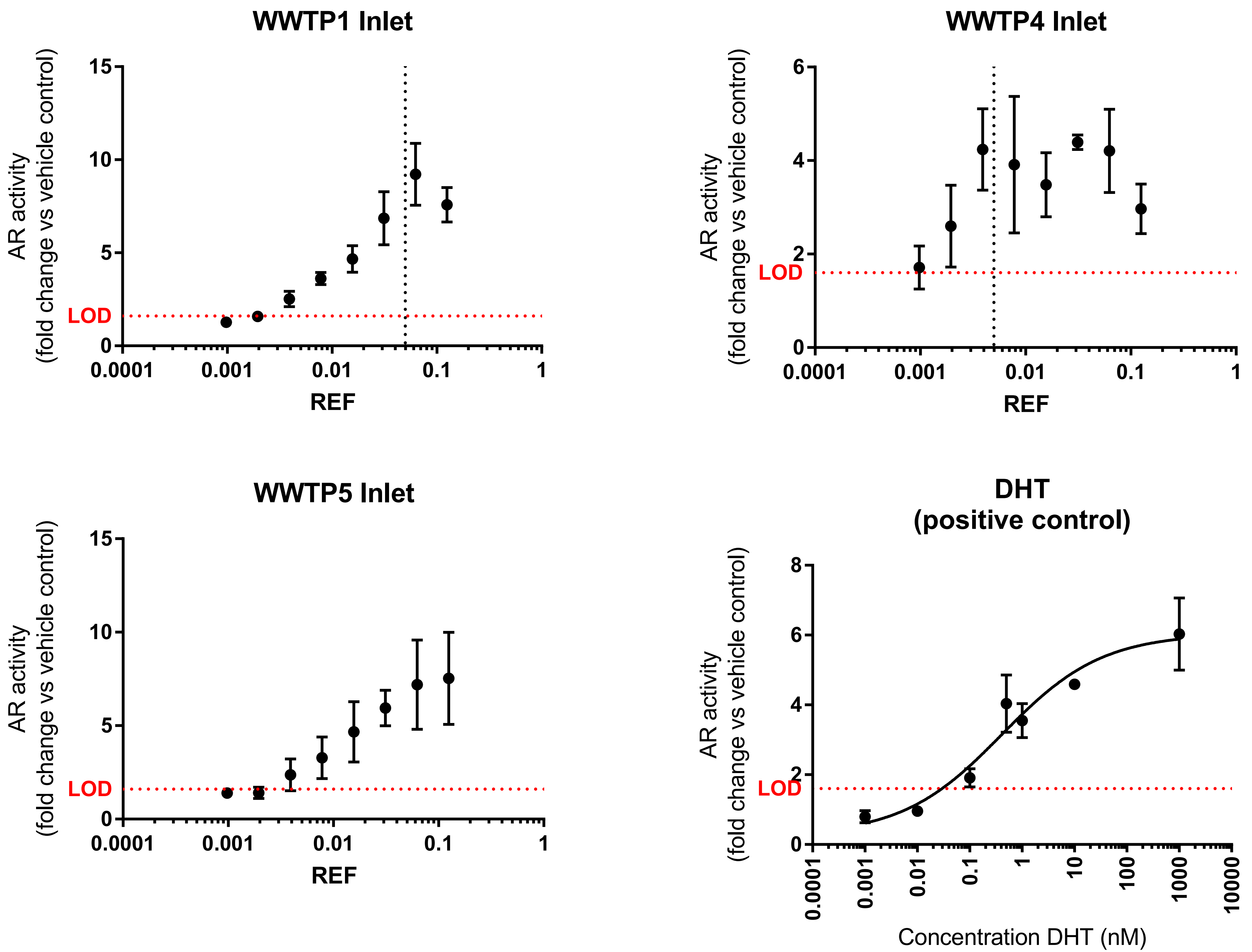
WWTP1 Inlet
AR activity
(fold change vs vehicle control)
15
10
5
0
LOD
0.0001
0.001
0.01
0.1
1
REF
WWTP4 Inlet
AR activity
(fold change vs vehicle control)
6
4
2
0
LOD
0.0001
0.001
0.01
0.1
1
REF
WWTP5 Inlet
AR activity
(fold change vs vehicle control)
15
10
5
0
LOD
0.0001
0.001
0.01
0.1
1
REF
DHT
(positive control)
AR activity
(fold change vs vehicle control)
8
6
4
2
0
LOD
0.0001
0.001
0.01
0.1
1
10
100
1000
10000
Concentration DHT (nM)

Figure SI-2D

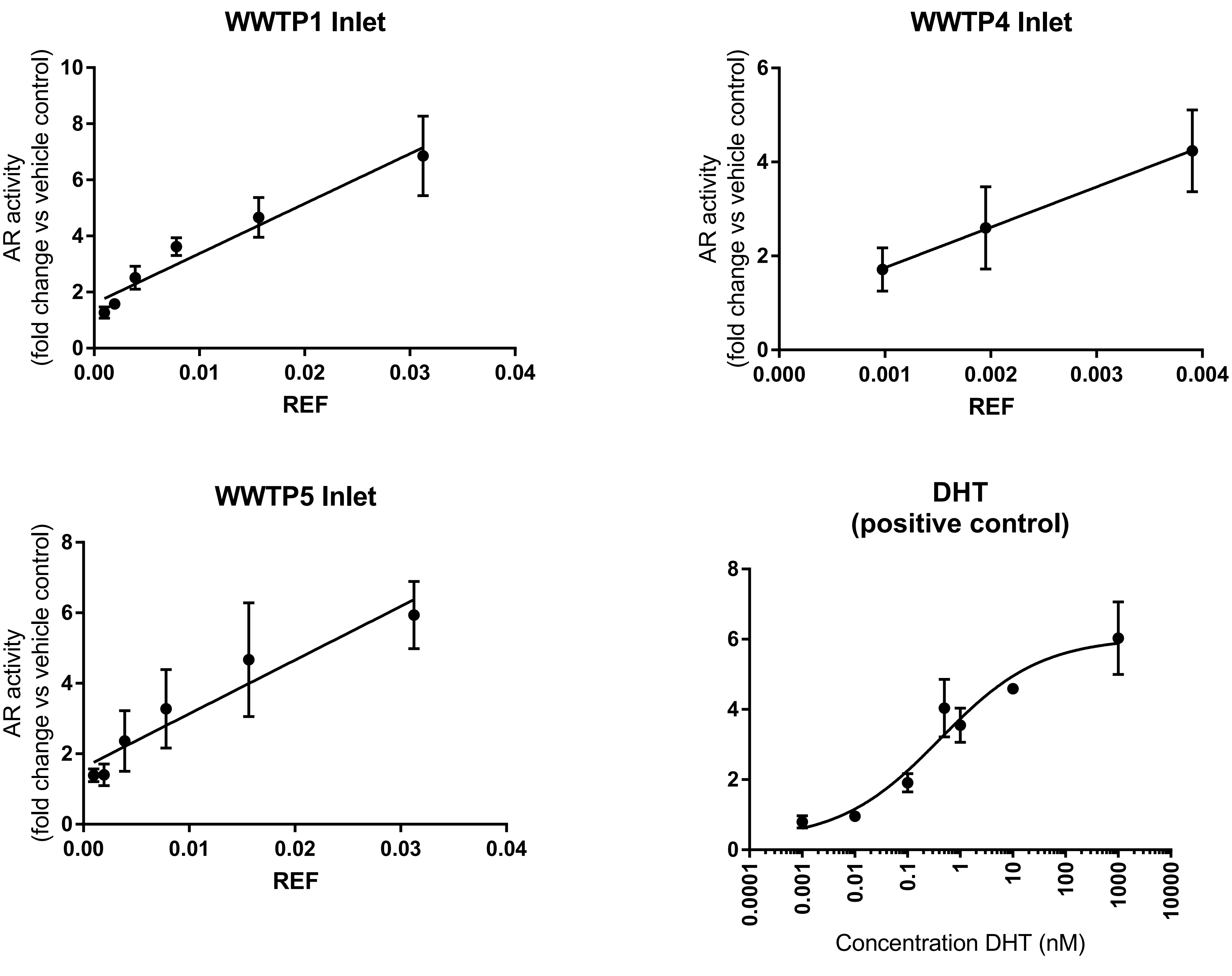

WWTP1 Inlet
AR activity
(fold change vs vehicle control)
10
8
6
4
2
0
0.00
0.01
0.02
0.03
0.04
REF
WWTP4 Inlet
AR activity
(fold change vs vehicle control)
6
4
2
0
0.000
0.001
0.002
0.003
0.004
REF
WWTP5 Inlet
AR activity
(fold change vs vehicle control)
8
6
4
2
0
0.00
0.01
0.02
0.03
0.04
REF
DHT
(positive control)
8
6
4
2
0
0.0001
0.001
0.01
0.1
1
10
100
1000
10000
Concentration DHT (nM)

Figure SI-2E

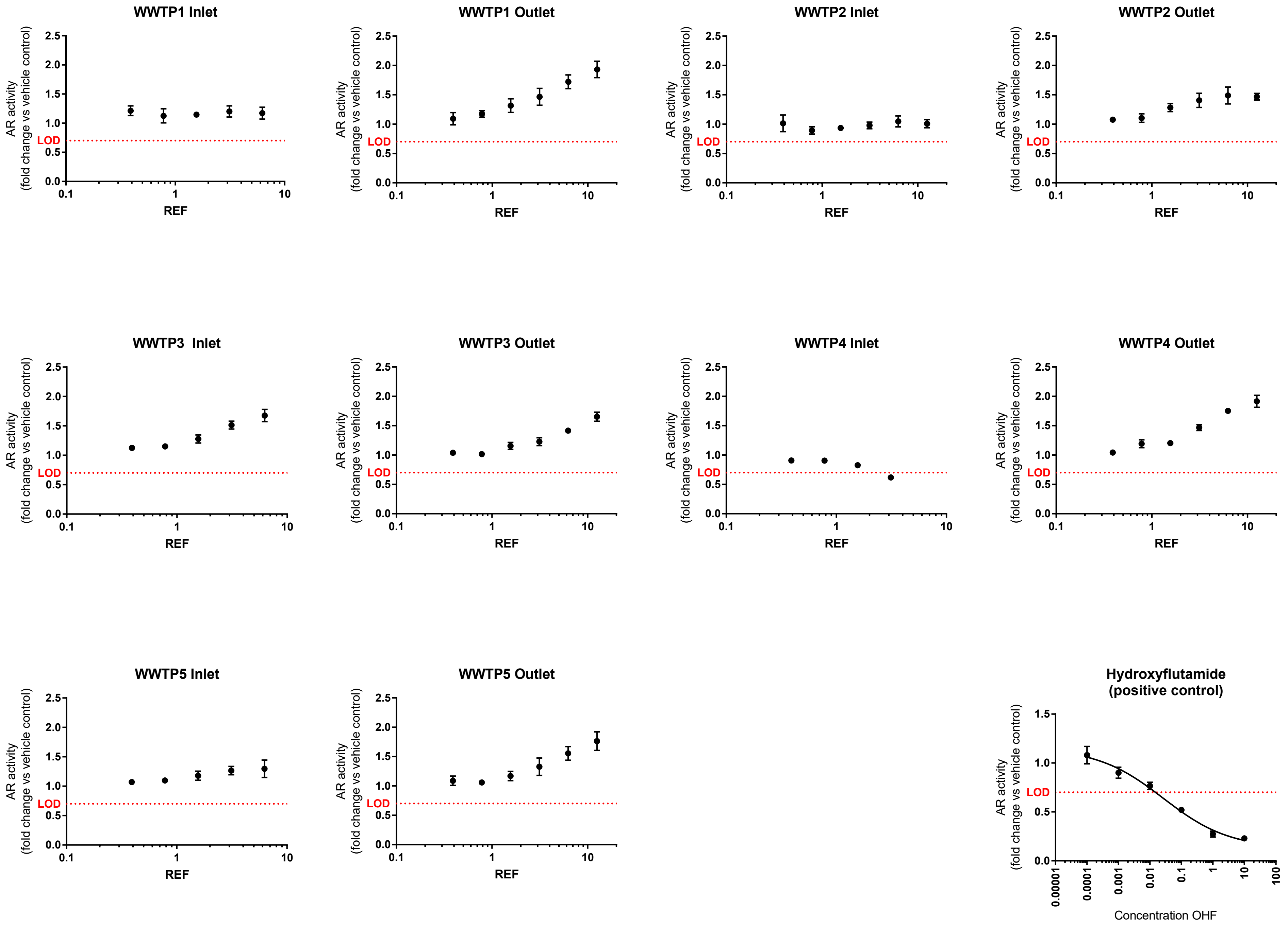

WWTP1 Inlet
WWTP1 Outlet
WWTP2 Inlet
WWTP2 Outlet
WWTP3 Inlet
WWTP3 Outlet
WWTP4 Inlet
WWTP4 Outlet
WWTP5 Inlet
WWTP5 Outlet
Hydroxyflutamide (positive control)
AR activity (fold change vs vehicle control)
LOD
REF
Concentration OHF

Figure SI-3A

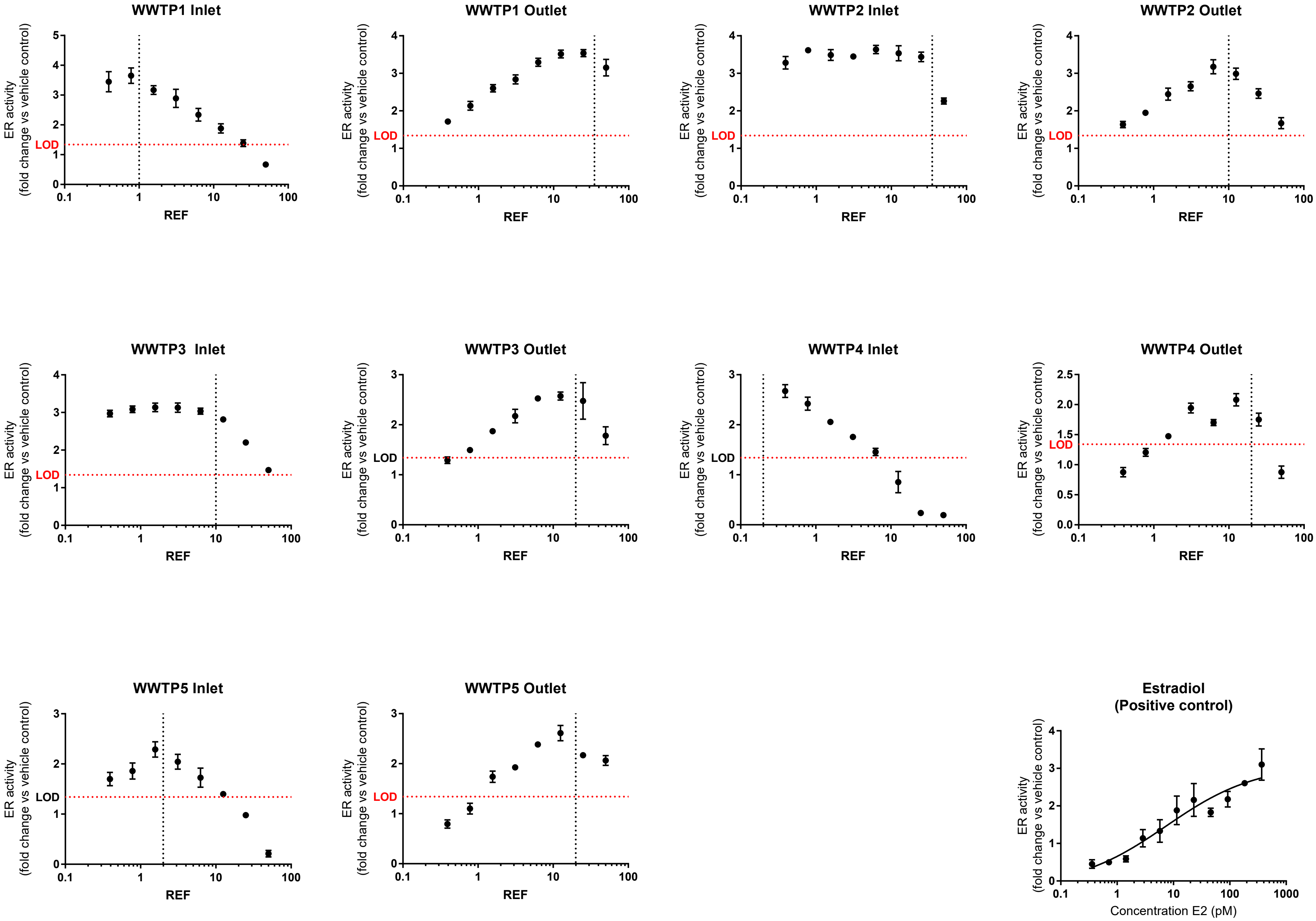
WWTP1 Inlet
WWTP1 Outlet
WWTP2 Inlet
WWTP2 Outlet
WWTP3 Inlet
WWTP3 Outlet
WWTP4 Inlet
WWTP4 Outlet
WWTP5 Inlet
WWTP5 Outlet
Estradiol
(Positive control)
ER activity
(fold change vs vehicle control)
LOD
REF
Concentration E2 (pM)
0.1
1
10
100
1000

Figure SI-3B

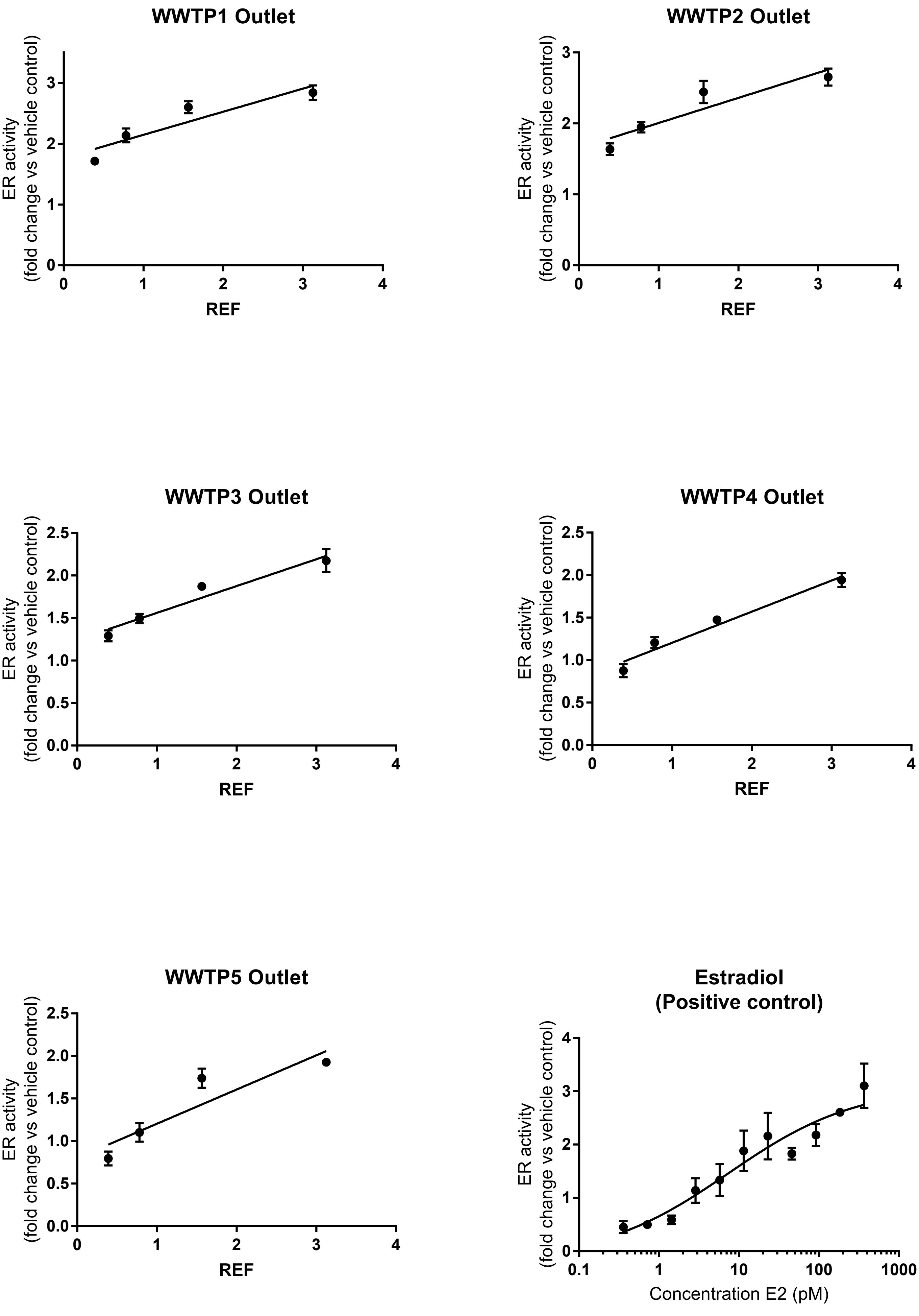
WWTP1 Outlet
WWTP2 Outlet
WWTP3 Outlet
WWTP4 Outlet
WWTP5 Outlet
Estradiol
(Positive control)
ER activity
(fold change vs vehicle control)
REF
0
1
2
3
4
0.0
0.5
1.0
1.5
2.0
2.5
0.1
10
100
1000
Concentration E2 (pM)

Figure SI-3C

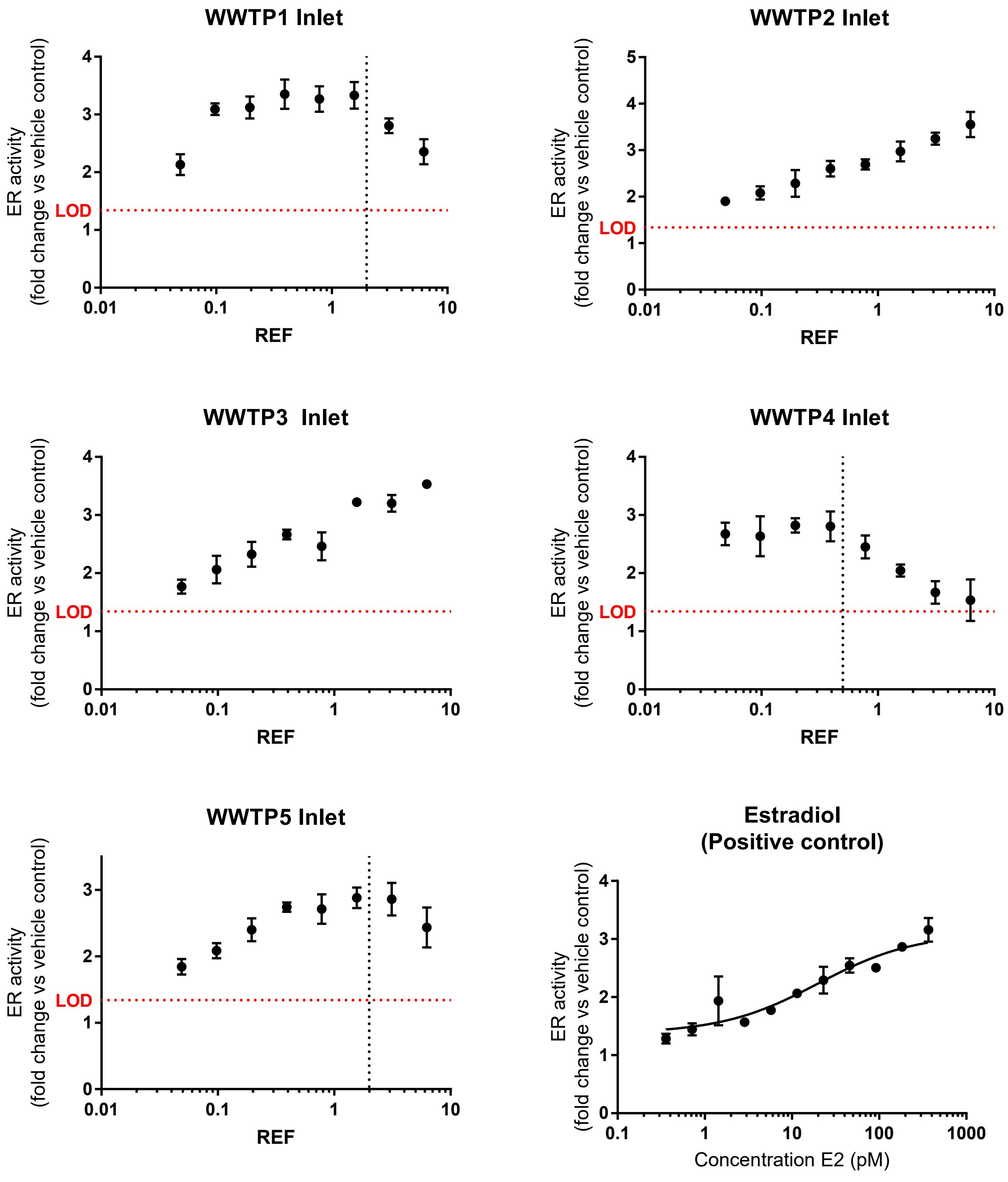
WWTP1 Inlet
ER activity
(fold change vs vehicle control)
LOD
0
1
2
3
4
0.01
0.1
1
10
REF
WWTP2 Inlet
ER activity
(fold change vs vehicle control)
LOD
0
1
2
3
4
5
0.01
0.1
1
10
REF
WWTP3 Inlet
ER activity
(fold change vs vehicle control)
LOD
0
1
2
3
4
0.01
0.1
1
10
REF
WWTP4 Inlet
ER activity
(fold change vs vehicle control)
LOD
0
1
2
3
4
0.01
0.1
1
10
REF
WWTP5 Inlet
ER activity
(fold change vs vehicle control)
LOD
0
1
2
3
0.01
0.1
1
10
REF
Estradiol
(Positive control)
ER activity
(fold change vs vehicle control)
0
1
2
3
4
0.1
1
10
100
1000
Concentration E2 (pM)

Figure SI-3D

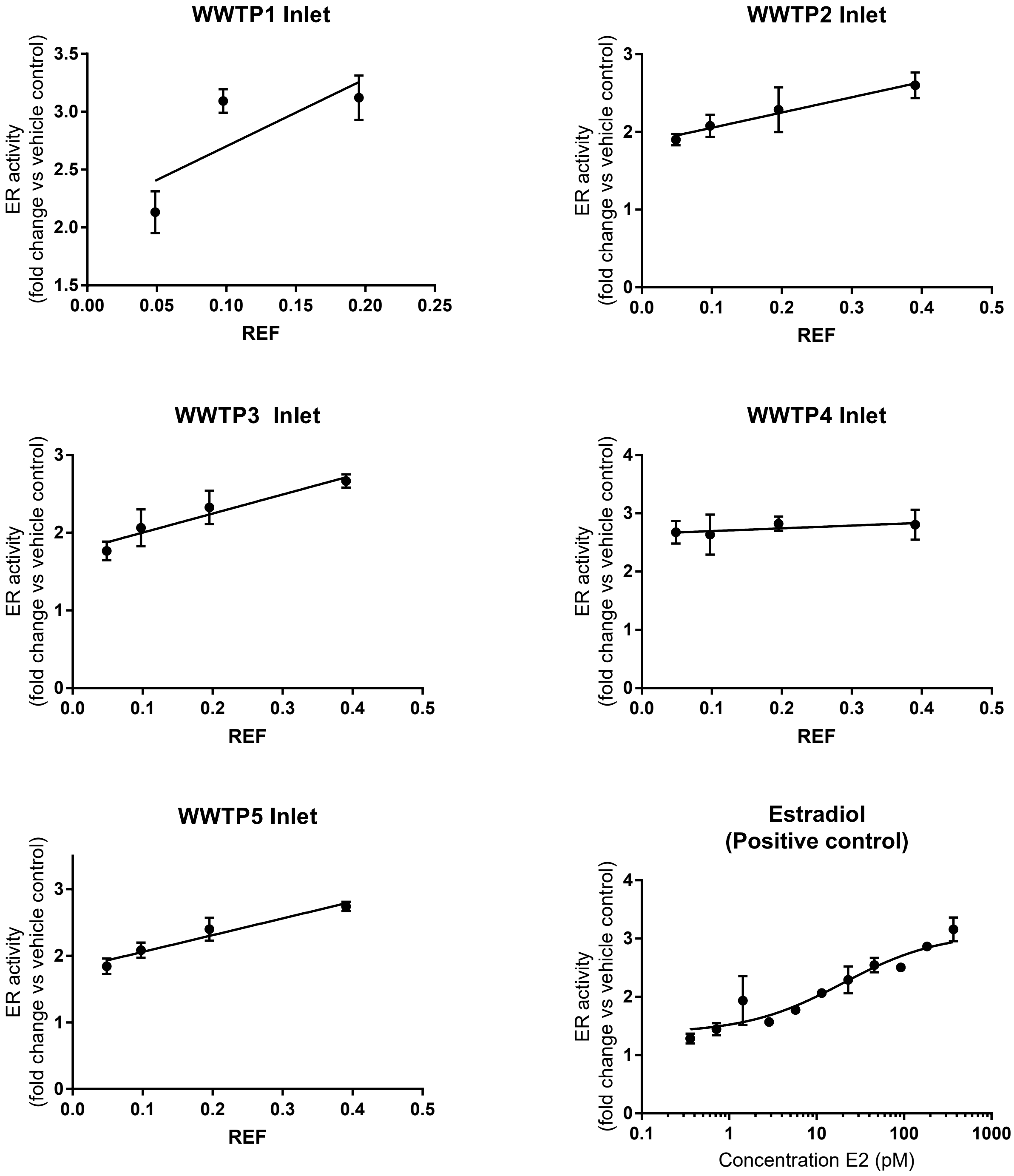
WWTP1 Inlet
ER activity
(fold change vs vehicle control)
REF
WWTP2 Inlet
WWTP3 Inlet
WWTP4 Inlet
WWTP5 Inlet
Estradiol
(Positive control)
Concentration E2 (pM)

Figure SI-3E

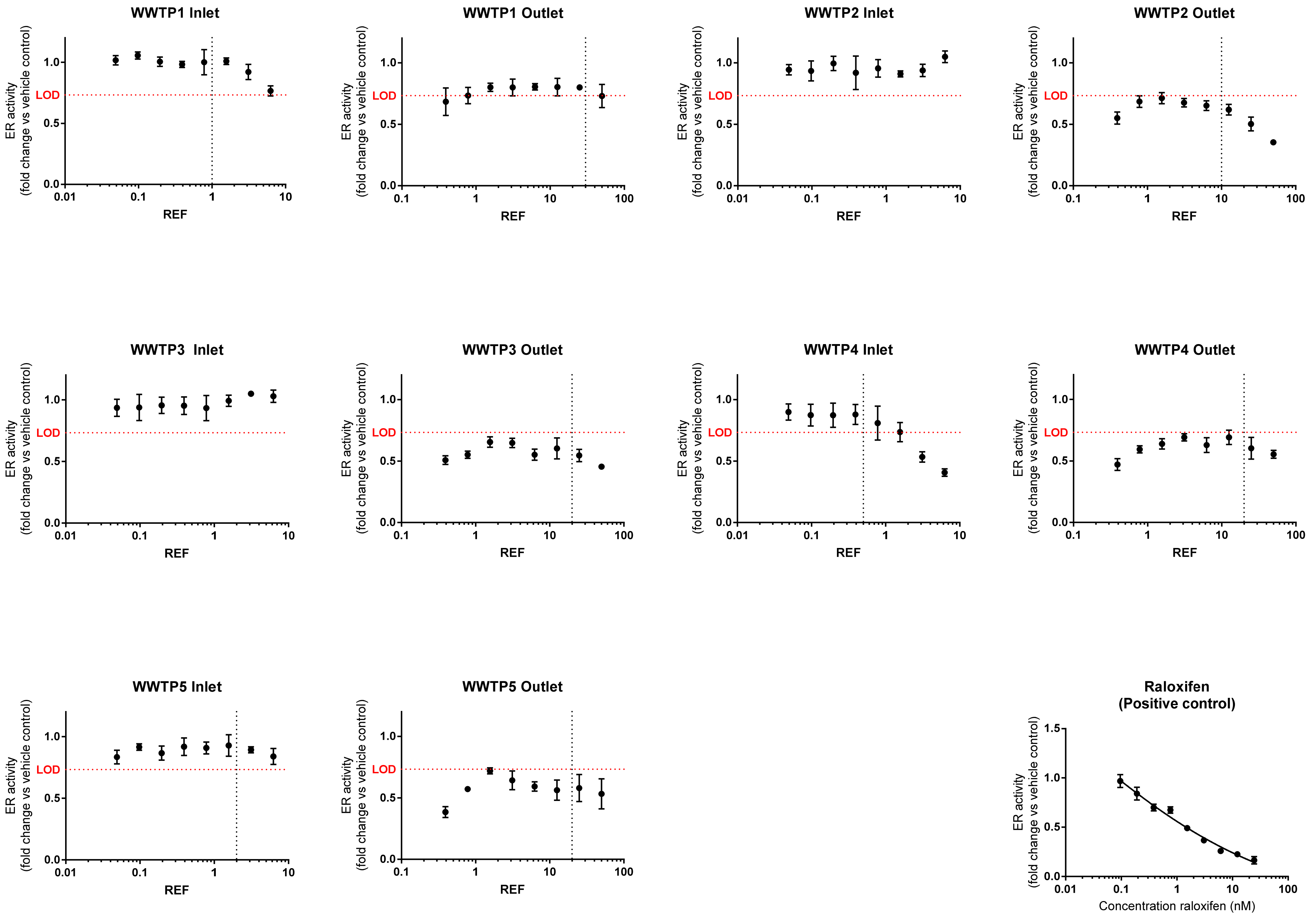

WWTP1 Inlet
WWTP1 Outlet
WWTP2 Inlet
WWTP2 Outlet
WWTP3 Inlet
WWTP3 Outlet
WWTP4 Inlet
WWTP4 Outlet
WWTP5 Inlet
WWTP5 Outlet
Raloxifen
(Positive control)
ER activity
(fold change vs vehicle control)
LOD
REF
Concentration raloxifen (nM)
0.0
0.5
1.0
1.5
0.01
0.1
1
10
100

Figure SI-4

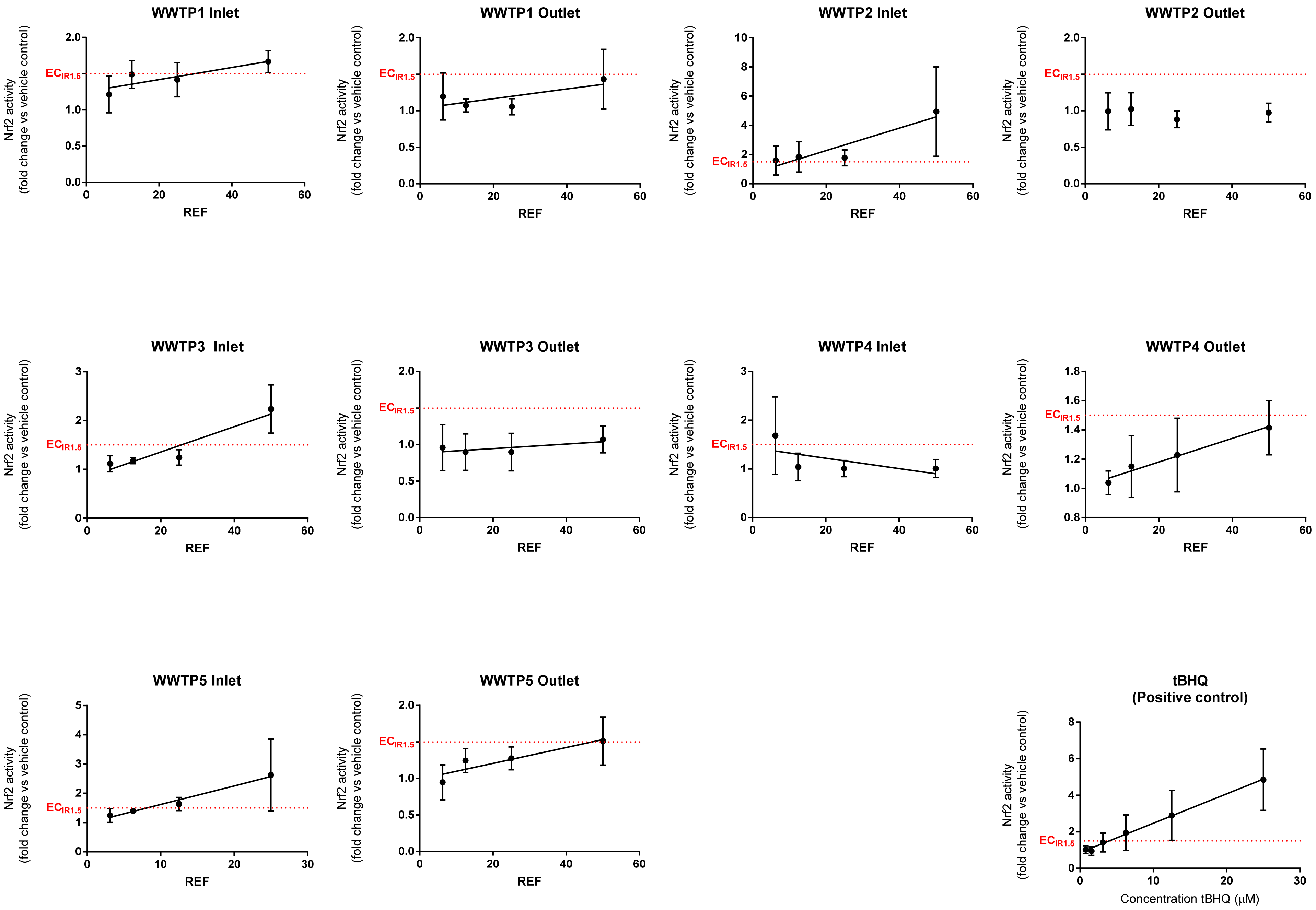
WWTP1 Inlet
WWTP1 Outlet
WWTP2 Inlet
WWTP2 Outlet
WWTP3 Inlet
WWTP3 Outlet
WWTP4 Inlet
WWTP4 Outlet
WWTP5 Inlet
WWTP5 Outlet
tBHQ
(Positive control)
Nrf2 activity
(fold change vs vehicle control)
REF
$EC_{IR1.5}$
Concentration tBHQ (μM)

Figure SI-5A

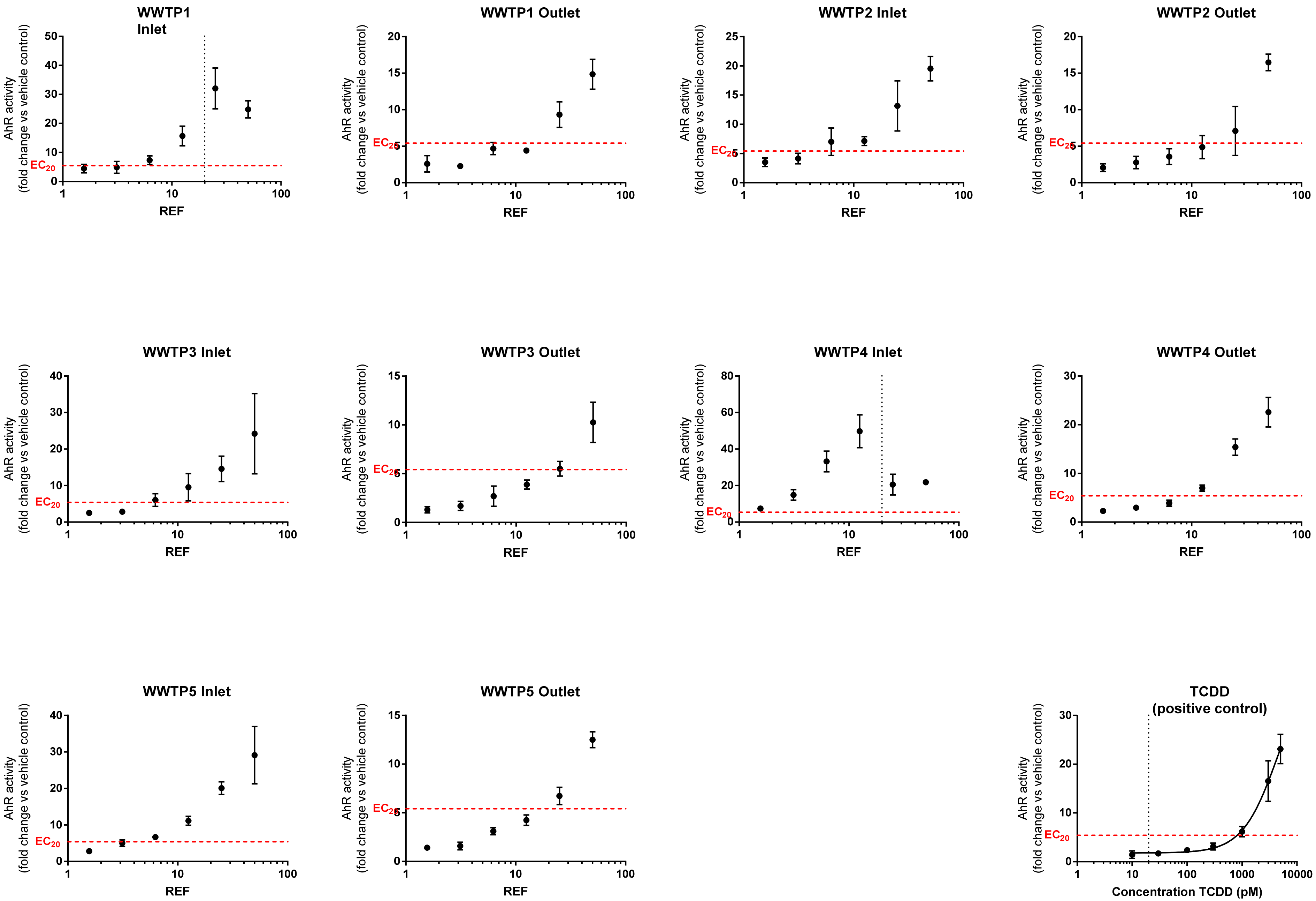

WWTP1 Inlet
WWTP1 Outlet
WWTP2 Inlet
WWTP2 Outlet
WWTP3 Inlet
WWTP3 Outlet
WWTP4 Inlet
WWTP4 Outlet
WWTP5 Inlet
WWTP5 Outlet
TCDD (positive control)
AhR activity (fold change vs vehicle control)
REF
Concentration TCDD (pM)
EC_{20}

Figure SI-5B

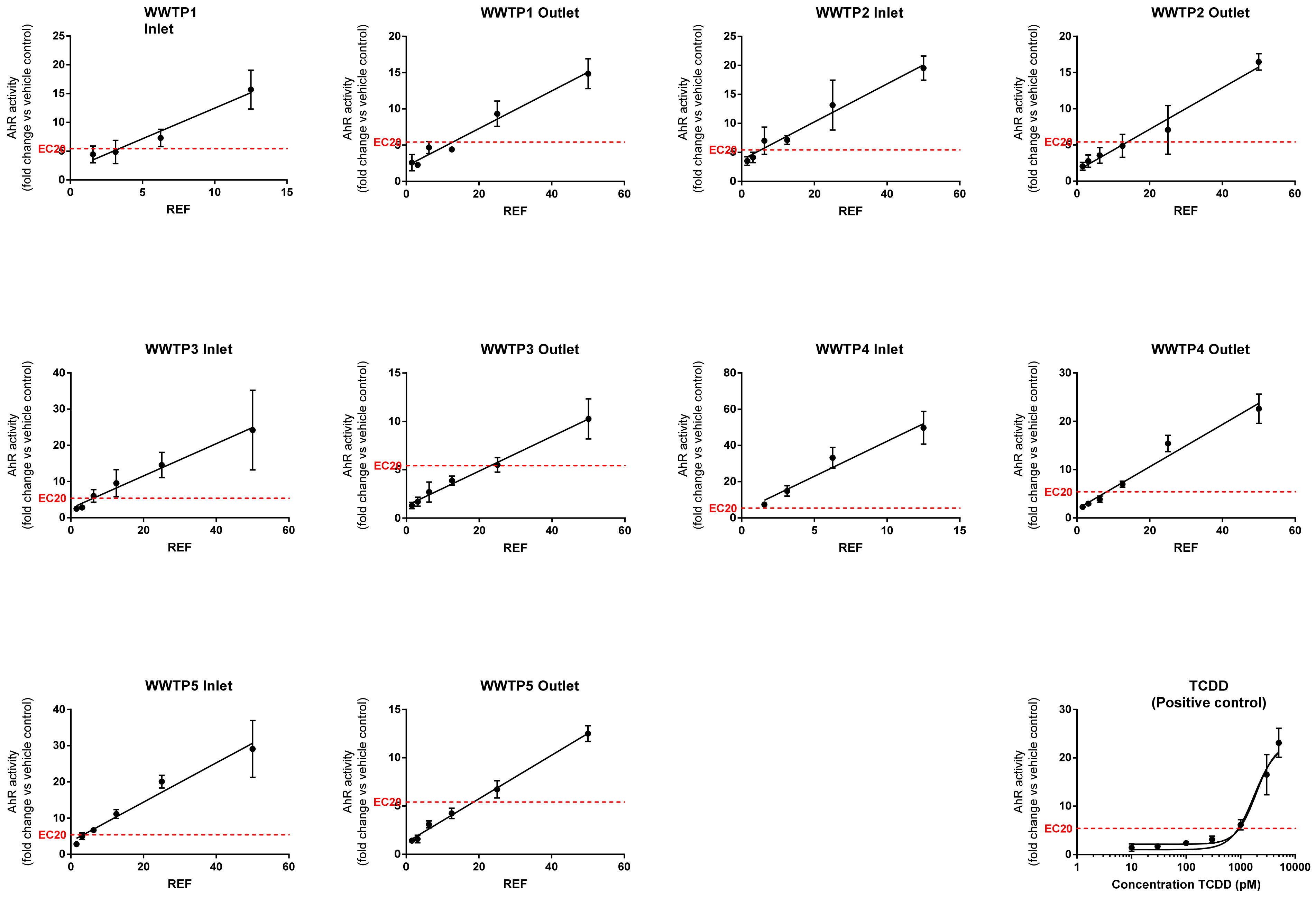
WWTP1 Inlet
WWTP1 Outlet
WWTP2 Inlet
WWTP2 Outlet
WWTP3 Inlet
WWTP3 Outlet
WWTP4 Inlet
WWTP4 Outlet
WWTP5 Inlet
WWTP5 Outlet
TCDD (Positive control)
AhR activity (fold change vs vehicle control)
REF
EC20
Concentration TCDD (pM)

Figure SI-6A

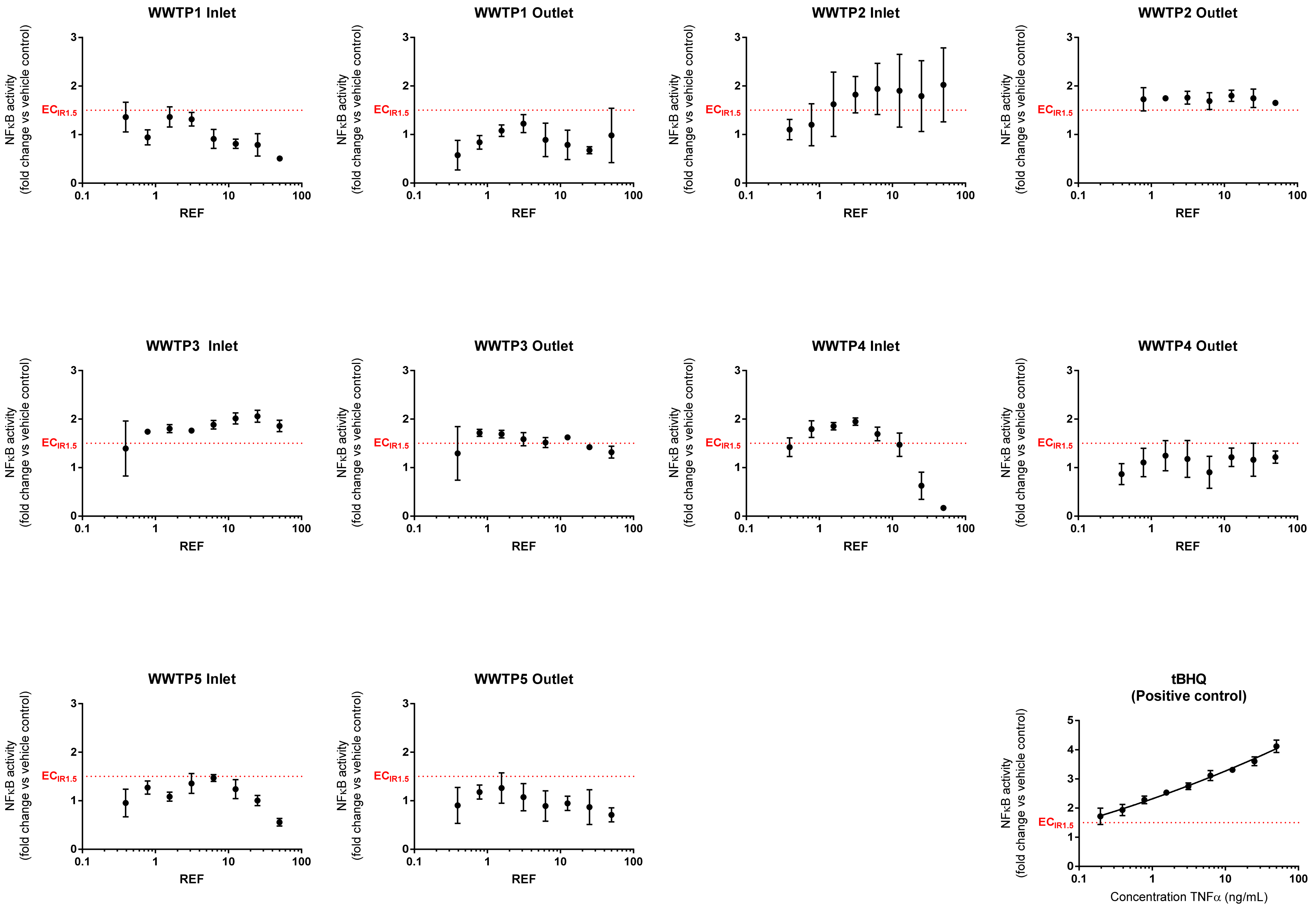
WWTP1 Inlet
WWTP1 Outlet
WWTP2 Inlet
WWTP2 Outlet
WWTP3 Inlet
WWTP3 Outlet
WWTP4 Inlet
WWTP4 Outlet
WWTP5 Inlet
WWTP5 Outlet
tBHQ
(Positive control)
NFκB activity
(fold change vs vehicle control)
EC$_{IR1.5}$
REF
Concentration TNFα (ng/mL)
0
1
2
3
4
5
0.1
1
10
100

Figure SI-6B

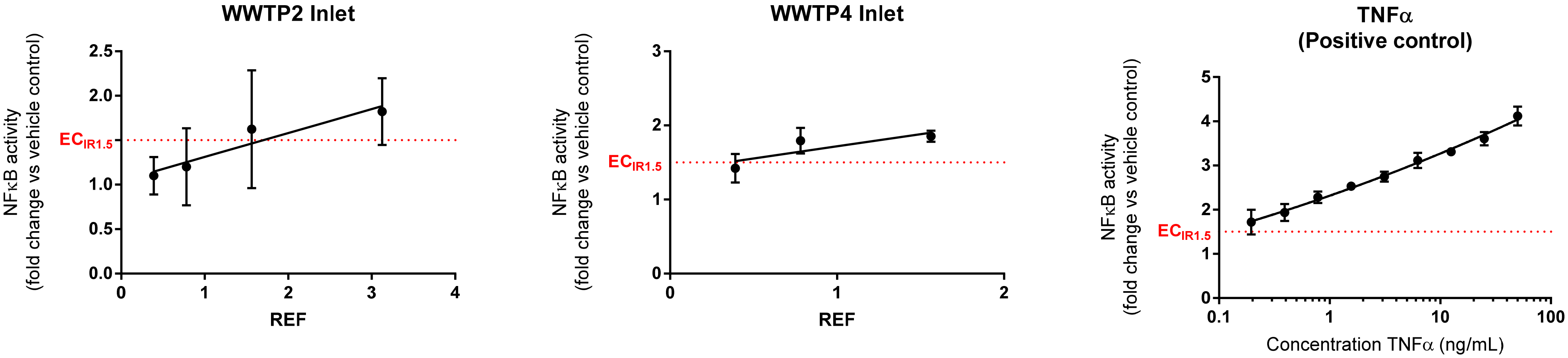
WWTP2 Inlet
NFκB activity
(fold change vs vehicle control)
EC$_{IR1.5}$
2.5
2.0
1.5
1.0
0.5
0.0
0
1
2
3
4
REF
WWTP4 Inlet
NFκB activity
(fold change vs vehicle control)
EC$_{IR1.5}$
3
2
1
0
0
1
2
REF
TNFα
(Positive control)
NFκB activity
(fold change vs vehicle control)
EC$_{IR1.5}$
5
4
3
2
1
0
0.1
1
10
100
Concentration TNFα (ng/mL)
